# Lectin-Based Substrate Detection in Fabry Disease Using the Gb3-Binding Lectins StxB and LecA

**DOI:** 10.3390/ijms26052272

**Published:** 2025-03-04

**Authors:** Serap Elçin-Guinot, Simon Lagies, Yoav Avi-Guy, Daniela Neugebauer, Tobias B. Huber, Christoph Schell, Bernd Kammerer, Winfried Römer

**Affiliations:** 1 Faculty of Biology, University of Freiburg, Schänzlestraße 1, 79104 Freiburg, Germany; serap.guinot@bioss.uni-freiburg.de (S.E.-G.); yoav.avi-guy@dkfz.de (Y.A.-G.); daniela.neugebauer@uniklinik-freiburg.de (D.N.); 2 BIOSS, Centre for Biological Signaling Studies, University of Freiburg, Schänzlestraße 18, 79104 Freiburg, Germany; bernd.kammerer@oc.uni-freiburg.de; 3 CIBSS, Centre for Integrative Biological Signaling Studies, University of Freiburg, Schänzlestraße 18, 79104 Freiburg, Germany; 4 Core Competence Metabolomics, Hilde-Mangold-Haus, University of Freiburg, Habsburgerstraße 19, 79104 Freiburg, Germany; simon.lagies@oc.uni-freiburg.de; 5 Institute of Organic Chemistry, University of Freiburg, Albertstraße 19, 79104 Freiburg, Germany; 6 III. Department of Medicine, University Medical Center Hamburg-Eppendorf (UKE), Martinistraße 52, 20246 Hamburg, Germany; t.huber@uke.de; 7 Hamburg Center for Kidney Health, University Medical Center Hamburg-Eppendorf (UKE), Martinistraße 52, 20246 Hamburg, Germany; 8 Faculty of Medicine, Institute for Surgical Pathology Medical Center, University of Freiburg, Breisacher Str. 115A, 70106 Freiburg, Germany; christoph.schell@uniklinik-freiburg.de; 9 Freiburg Institute for Advanced Studies (FRIAS), University of Freiburg, Albertstraße 19, 79106 Freiburg, Germany; 10 Spemann Graduate School of Biology and Medicine (SGBM), University of Freiburg, Albertstraße 19, 79104 Freiburg, Germany

**Keywords:** lysosomal storage disorder, Gb3, lyso-Gb3, glycosphingolipids, lectins, StxB, LecA, anti-Gb3 antibody, patient-derived Fabry fibroblasts, *GLA*-knockout human podocytes

## Abstract

Fabry disease, the second most common lysosomal storage disorder, is caused by a deficiency of α-galactosidase A (α-Gal A), which leads to an accumulation of glycosphingolipids (GSL), mainly globotriaosylceramide (also known as Gb3). This aberrant GSL metabolism subsequently causes cellular dysfunction; however, the underlying cellular and molecular mechanisms are still unknown. There is growing evidence that damage to organelles, including lysosomes, mitochondria, and plasma membranes, is associated with substrate accumulation. Current methods for the detection of Gb3 are based on anti-Gb3 antibodies, the specificity and sensitivity of which are problematic for glycan detection. This study presents a robust method using lectins, specifically the B-subunit of Shiga toxin (StxB) from *Shigella dysenteriae* and LecA from *Pseudomonas aeruginosa*, as alternatives for Gb3 detection in Fabry fibroblasts by flow cytometry and confocal microscopy. StxB and LecA showed superior sensitivity, specificity, and consistency in different cell types compared to all anti-Gb3 antibodies used in this study. In addition, sphingolipid metabolism was analyzed in primary Fabry fibroblasts and α-Gal A knockout podocytes using targeted tandem liquid chromatography-mass spectrometry. Our findings establish lectins as a robust tool for improved diagnostics and research of Fabry disease and provide evidence of SL changes in cultured human cells, filling a knowledge gap.

## 1. Introduction

Fabry disease (FD) is an X-linked lysosomal storage disorder caused by several mutations that impair the activity of the lysosomal hydrolase α-galactosidase A (α-Gal A)**.** α-Gal A initiates the degradation of glycoconjugates, mainly the glycosphingolipid globotriaosylceramide (Galα1-4Galβ1-4Glc-ceramide, also known as Gb3) [1,2]. The deficiency or dysfunction of α-Gal A leads to progressive accumulation of Gb3 and its derivatives, such as globotriaosylsphingosine (lyso-Gb3, a water-soluble deacylated form of Gb3) in lysosomes, ultimately resulting in cellular damage affecting various cell types and tissues, including endothelial cells, podocytes, and cardiomyocytes [3]. This accumulation disrupts normal cellular function, leading to a broad spectrum of clinical manifestations ranging from angiokeratomas, hypohidrosis, and neuropathic pain to renal failure, cardiac hypertrophy, and cerebrovascular complications [4,5]. To date, several cellular mechanisms underlying Fabry pathogenesis have been proposed in the literature, including ion-channel sensitization [6], structural and functional mitochondrial dysfunction [7,8], and impaired protein synthesis [9], with the aim of elucidating determinants beyond substrate accumulation. In a recent study, the accumulation of α-synuclein (SNCA) in lysosomes, independent of Gb3 accumulation, was identified as another factor contributing to lysosomal dysfunction. Pharmacological inhibition of SNCA has been shown to restore lysosomal structure-function dynamics, clearly indicating one of many factors leading to Fabry pathogenesis [10]. There are currently two therapies for Fabry disease, enzyme replacement therapy (ERT) and chaperone therapy [11]. ERT is the only treatment available for Fabry patients, which is not restricted by the type of mutation. It helps to prevent the progression of the disease and only offers a significant clinical benefit if it is initiated at an early stage of disease development. On the other hand, chaperone therapy is limited to certain mutations and is therefore only suitable for a subset of patients [12]. While further developments in treatments are underway, such as substrate reduction, mRNA, and gene therapies, it remains critical to gain a deeper understanding of the underlying cellular mechanisms by which substrate accumulation leads to cell dysfunction [13,14].

Several studies have focused on monitoring the accumulation of Gb3 in body fluids such as blood and urine, which are considered crucial for assessing the efficacy of therapeutic approaches [15,16]. In addition, another substrate, lyso-Gb3, was found to correlate with disease severity, providing valuable insight into its potential role as a biomarker for treatment response [3]. The latest results of a gene therapy study with lentiviruses further support this research [17].

Antibodies are currently considered the gold standard for methods to identify glycolipids such as Gb3 and related substrates in immunoassays or imaging techniques [18,19,20]. Although anti-Gb3 antibodies provide valuable insights into Gb3 detection in most cases, their function is limited by several constraints. Firstly, the specificity and sensitivity of anti-Gb3 antibodies can vary greatly, as glycolipids are generally not as strong antigens as proteins. Several glycolipid antibodies cross-react and display poly-specificity during the recognition process of pathogens by the immune system, leading to autoimmune diseases, such as lipid A [21] or the recognition of self-glycans [22]. Secondly, glycolipids such as Gb3 are embedded in the complex matrix of the plasma membrane, where the surrounding lipid environment influences the presentation of the carbohydrate head group. This poses a challenge for antibody binding, as the recognition of the epitope may depend on conformations that are not consistently accessible [23,24,25]. The lack of robust detection methods limits our ability to fully understand the dynamics of Gb3 accumulation and its role in Fabry disease pathology. In this context, lectins could serve as an excellent tool for the detection of Gb3 and other glycoconjugates. Lectins are non-catalytic, carbohydrate-binding proteins, some of which have high specificity for glycan structures. They can be regarded as natural decoders of glycans. Since they usually assemble as oligomers, they can interact with several binding partners simultaneously, which significantly increases their avidity through hetero-multivalent binding, as in the case of the B-subunit of cholera toxin [26]. This inherent specificity makes them ideal candidates for studying glycoconjugates from a different angle [27]. Furthermore, lectins can provide a more refined understanding of glycosphingolipid compositions as they are able to distinguish between closely related glycans based on subtle structural and environmental differences [28].

In this article, we focus mainly on the evaluation of two lectins, namely the homo-pentameric B-subunit of Shiga toxin (StxB) from *Shigella dysenteriae* and the homo-tetrameric LecA from *Pseudomonas aeruginosa*, for their ability to detect Gb3 and related glycans that accumulate in Fabry fibroblasts. Shiga toxins are characterized by the general AB5 structure. StxB is the non-toxic, receptor-binding subunit of Shiga toxin. StxB enters cells by binding to Gb3 with high affinity, utilizing clathrin-dependent and clathrin-independent endocytosis pathways [29]. Upon ligand binding, StxB induces negative membrane curvature through Gb3 clustering, leading to the formation of tubular plasma membrane invaginations [30,31,32]. StxB bears three binding sites per monomer, i.e., it could in principle bind up to 15 Gb3. Mutational analysis revealed that binding sites 1 and 2 are crucial for the high affinity binding for Gb3, while site 3 has a rather low affinity for Gb3. Overall, the multivalency of the homo-pentameric structure, in which all binding sites point in the same direction towards the membrane, could be responsible for the improved avidity of StxB to Gb3 [29]. In contrast, LecA is a virulence factor of *P. aeruginosa* that has a high affinity for terminal Galα1,4-conjugates (mainly Gb3) and a lower affinity for glycans with terminal Galα-1,3 and Galα-1,6 conjugates [33]. Upon binding to Gb3, LecA triggers bacterial invasion of host cells through Gb3 clustering, a mechanism we have termed “lipid zipper”, and induction of downstream signaling pathways [34,35,36].

We demonstrate that the lectins StxB and LecA outperform conventional anti-Gb3 antibodies in terms of specificity, sensitivity, and consistency in different cell types. We present a comprehensive comparison of StxB and LecA with an anti-Gb3 antibody to evaluate their performance in Fabry fibroblasts by flow cytometry and confocal microscopy. In addition, we present the impaired glycosphingolipid metabolism in Fabry disease from primary human fibroblasts and human podocytes with knocked-out *GLA* using targeted liquid chromatography-mass spectroscopy (LC-MS). Ultimately, we pursue to establish lectins as a valuable, robust alternative or complementary tool to antibody-based methods, paving the way for research methods and improved diagnostics in Fabry disease.

## 2. Results

### 2.1. The Commercially Available Anti-Gb3 Antibodies Tested Do Not Recognize Gb3 in the Plasma Membrane of Fabry Fibroblasts

**Figure 1:**
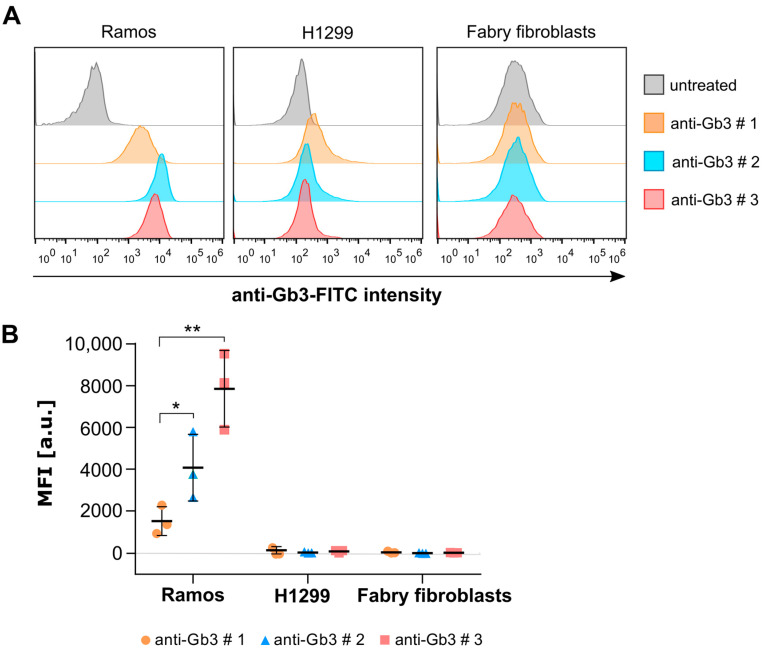
Binding of commercially available anti-Gb3 antibodies (FITC-labeled) was tested in various cell types. (**A**) Representative flow cytometry histograms of Ramos B cells, H1299 lung epithelial cells, and Fabry fibroblasts incubated with three different anti-Gb3 antibodies. (**B**) The mean fluorescence intensity values were calculated from the flow cytometry histograms as the geometric mean of the fluorescence intensity (MFI) of individually gated populations and normalized to the autofluorescence of each line (untreated). An unpaired, one-way ANOVA test was applied to test the significance of the data (*n* = 3). Error bars are means ± standard deviations (SD). Only significant values are displayed. * *p* < 0.05, ** *p* < 0.01.

Until now, anti-Gb3 antibodies were the only tools available to study Gb3 in Fabry disease research [37,38]. To evaluate the binding efficiency of anti-Gb3 antibodies, three different commercially available anti-Gb3 antibodies (tagged with FITC) were tested across three human cell types: Ramos B-cell lymphoma, H1299 lung epithelial carcinoma cells, and primary Fabry fibroblast cells. Flow cytometry analysis was performed to assess the binding of these antibodies to Gb3 on the plasma membrane of these cells. Ramos and H1299 cells were selected as positive controls for this experiment since their Gb3 content has already been analyzed by mass spectrometry in former studies [35,39], and Ramos cells have been recommended by the manufacturer for the validation of antibody binding. All three cell types were treated with antibodies (1:33 dilution) for 30 min on ice to ensure the specific binding of the antibodies to Gb3 on the plasma membrane (PM) while preventing any endocytic activity. In addition, Annexin V (PE-labeled) was used to stain and distinguish apoptotic cells and cells with disrupted membrane integrity from the healthy cell population. Figure 1A shows representative flow cytometry histograms of Ramos cells, H1299 cells, and Fabry fibroblasts, respectively. All three anti-Gb3 antibodies exhibited a noticeable increase in mean fluorescence intensity (from FITC tags) in Ramos cells compared to the auto-fluorescence levels of untreated cells, confirming successful binding to this cell line. On the contrary, only the binding of anti-Gb3 #1 was slightly distinguishable from the untreated control in H1299 cells. In Fabry fibroblasts, no FITC signal was detectable above the measured auto-fluorescence. The FITC signals were calculated as the geometric mean fluorescence intensity (MFI) and plotted as a bar graph (Figure 1B) showing significant differences in the MFI values recorded from each antibody in Ramos cells compared to untreated control, whereas H1299 cells and Fabry fibroblasts displayed no significant differences in any case. These results indicate that the antibodies were able to bind to Gb3 on the plasma membrane of Ramos cells to varying degrees; however, they were surprisingly unable to bind to either H1299 cells or Fabry fibroblasts.

### 2.2. Plasma Membrane and Whole Cell Gb3 Levels of Fabry and Healthy Fibroblasts Analyzed by Flow Cytometry Using Lectins

**Figure 2:**
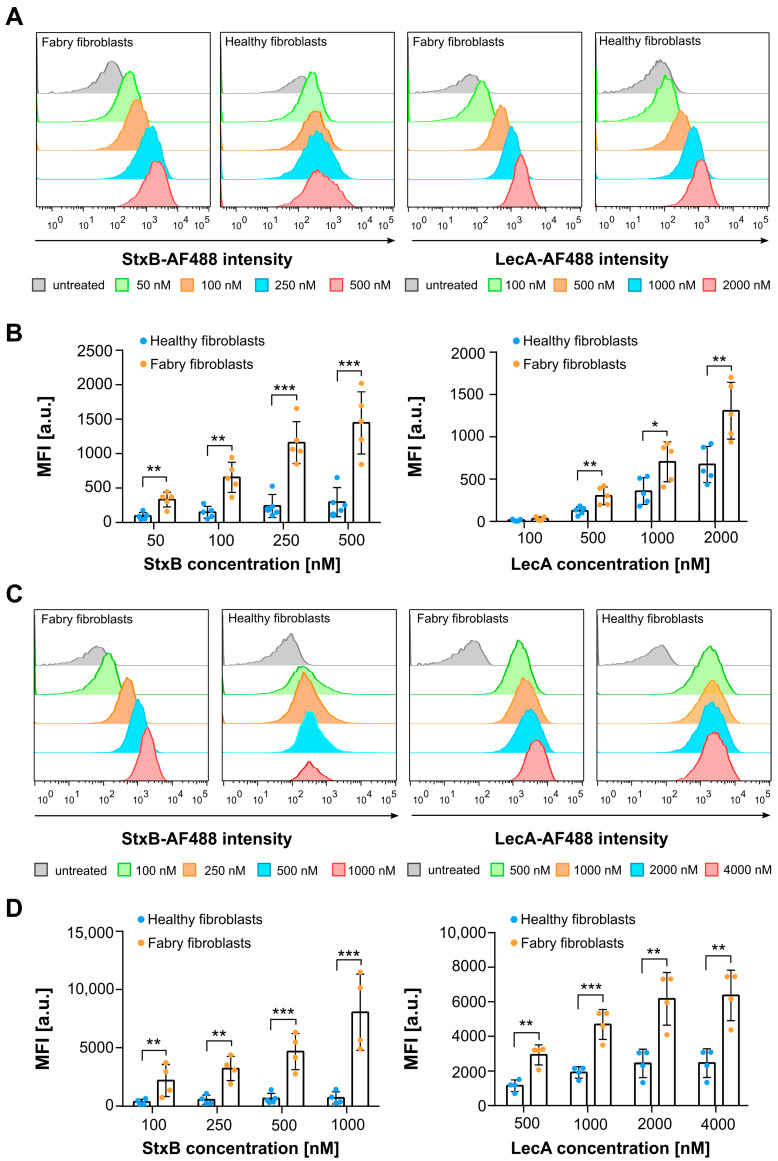
Fabry fibroblasts display a higher fluorescence signal of StxB-AF488 and LecA-AF488 compared to healthy fibroblasts in plasma membrane and whole cell flow cytometry analysis. (**A**) Representative histograms exhibit the mean fluorescence intensity from the plasma membrane of Fabry fibroblasts (left panel) and healthy fibroblasts (right panel) at varying concentrations of StxB-AF488 (50 nM, 100 nM, 250 nM, and 500 nM) and LecA-AF488 (100 nM, 500 nM, 1000 nM, and 2000 nM). (**B**) The MFI values were calculated from flow cytometry histograms (as depicted in (**A**)) as the geometric mean of the fluorescence intensity of individually gated populations and normalized to the auto-fluorescence of each cell type (untreated). (**C**) Representative histograms display the mean fluorescence intensity arising from the whole cell content of Fabry fibroblasts (left panel) and healthy fibroblasts (right panel) at varying concentrations of StxB-AF488 (100 nM, 250 nM, 500 nM, and 1000 nM) and LecA-AF488 (500 nM, 1000 nM, 2000 nM, and 4000 nM). (**D**) The MFI values were calculated from flow cytometry histograms (as depicted in (**C**)) as the geometric mean of the fluorescence intensity of individually gated populations and normalized to the auto-fluorescence of each type (untreated). An unpaired, two-tailed, parametric *t*-test was applied to test the significance of the data *(n* ≥ 3). Error bars are means ± SD. Only significant values are displayed. * *p* < 0.05, ** *p* < 0.01, *** *p* < 0.001.

The two lectins used in this study, StxB and LecA, are excellent candidates for Gb3 detection, particularly in Fabry disease cells, as they both intrinsically and specifically bind to Gb3. StxB selectively binds to Gb3, whereas LecA binds to Gb3 and potentially other glyco-conjugates containing terminal α-galactosyl residues. Flow cytometry experiments were performed on Fabry cells using StxB and LecA, labeled with Alexa Fluor 488 dyes (abbreviated from now on as AF488). According to the same experimental design as the anti-Gb3 antibody assays, initially only the plasma membranes of Fabry and healthy fibroblasts were targeted (Figure 2A,B). An important difference between the Fabry and healthy fibroblasts was the formation of aggregates after detachment of the cells by EDTA treatment. The healthy cells formed many aggregates in the cell suspension, which was only the case to a very small extent in the Fabry cells. As a downstream effect of aggregate formation, we counted fewer cells in the final population and observed more apoptotic cells in healthy fibroblasts (see Supplementary Figure S1). In the case of both lectins, Fabry cells show a remarkable increase in fluorescence signal intensity with increasing lectin concentration, indicating increased binding of lectins (Figure 2A). It is important to note that Fabry cells’ signal intensity saturated between 250 nM and 500 nM StxB treatment (Figure 2A, left panel, and Figure 2B. However, no saturation of binding was observed with the lectin LecA, even at a concentration of 2000 nM (Figure 2A, right panel, and Figure 2B). Healthy fibroblasts did not show a drastic increase in fluorescence signal intensity with increasing concentration of StxB, indicating that Gb3 occurs only marginally in these cells. On the contrary, increasing the concentration of LecA led to an increase in fluorescence signal intensity in healthy fibroblasts as well (Figure 2A,B). The StxB signal arising from Fabry fibroblasts was significantly higher compared to healthy fibroblasts at given lectin concentrations due to the early saturation of binding in healthy fibroblasts. The LecA signal of Fabry fibroblasts was also significantly higher than that of healthy fibroblasts; however, the differences in the LecA signal were less pronounced than for StxB (Figure 2B). Nevertheless, these results clearly demonstrate that both lectins successfully bind to the plasma membrane of Fabry and, to a lower extent, healthy fibroblasts, providing valuable insights into both cell lines.

In a complementary experiment, we have used both lectins, StxB and LecA, to stain the respective receptors in both cell types (healthy and Fabry fibroblasts) on a whole cell level (Figure 2C,D), not only at the plasma membrane. In this experiment, cells were detached with trypsin-EDTA and collected, fixed with a 4% PFA solution, and permeabilized with a 0.2% saponin solution prior to the lectin treatment. Cells were treated with both lectins at room temperature at given concentrations for one hour and then were subjected to flow cytometry. The healthy fibroblasts followed a similar trend to the results for the evaluation of the plasma membrane in these cells. StxB and LecA signals quickly reached saturation in healthy fibroblasts at concentrations of 250 nM to 1000 nM, respectively (Figure 2C). Overall, the use of both lectins reveals a significant difference in fluorescence signal intensity in Fabry fibroblasts, with StxB showing a detectable signal up to 1000 nM (Figure 2C, left panel) and LecA up to 2000 nM (Figure 2C, right panel) with a slow approach to saturation. This suggests that at least double the concentration of both lectins is required to effectively target the increased Gb3 content when analyzing whole cells compared to the plasma membrane of fibroblasts. In whole cell analysis, StxB showed a signal six times higher than at the plasma membrane only, while it was five times higher for LecA (Figure 2D).

In summary, the use of lectins as a tool to study Fabry cells provides an excellent perspective on the accumulating substrates in Fabry cells. While commercially available anti-Gb3 antibodies failed to show a reasonable signal when analyzing the plasma membrane of Fabry fibroblasts, StxB and LecA were able to bind to Fabry and healthy fibroblasts, allowing detailed analysis of these cell lines.

### 2.3. Lectins Exhibit Advanced Binding Kinetics in Fabry Fibroblasts Compared to an Anti-Gb3 Antibody in Immunofluorescence Assays

**Figure 3:**
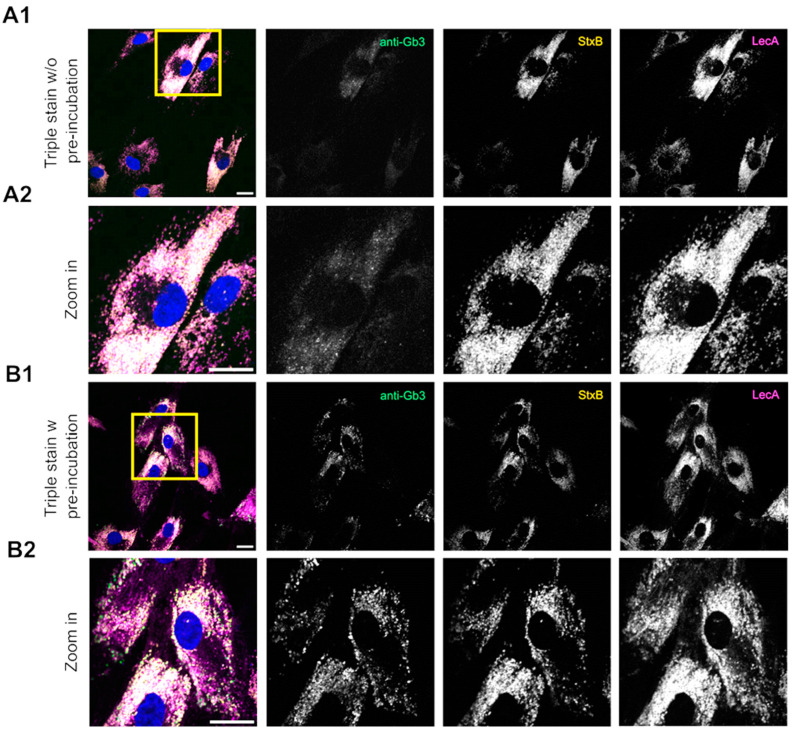
Triple stain of Fabry fibroblasts using an anti-Gb3 antibody, StxB, and LecA. Representative confocal images of Fabry fibroblasts are depicted, which were stained with the anti-Gb3 antibody # 2 (1:20), without (**A1**,**A2**) or with pre-incubation (**B1**,**B2**), then stained with a secondary antibody-AF488 (1:200) against anti-Gb3, and StxB-AF555 (1:100), and LecA-AF647 (1:200). (**A1**,**B1**) display the full view of the merged image (2048 × 2048 pixels) and grayscale images of individual channels. Zoomed in areas of the merge from (**A1**,**B1**) (indicated by a yellow square) and the grayscale images are presented in (**A2**,**B2**). (**A1**,**A2**) In the absence of pre-incubation of the anti-Gb3 antibody, i.e., simultaneous administration of all three tools, the anti-Gb3 antibody exhibited a very weak signal. (**B1**,**B2**) The anti-Gb3 antibody demonstrated a detectable signal when it was pre-incubated ahead of the lectins, followed by a co-incubation of the lectins with the secondary antibody against anti-Gb3. All scale bars represent 20 µm.

Due to the inconsistencies observed in anti-Gb3 staining during flow cytometry experiments (poor cell type-specific binding), we decided to perform a comparative analysis of lectins and anti-Gb3 antibodies by investigating their binding kinetics using an immunofluorescence assay. Hence, we used the primary anti-Gb3 antibody #2 (which was subsequently incubated with a secondary AF488-labeled antibody), StxB-AF555, and LecA-AF647 in a comparative manner to stain Gb3 in fibroblasts. We called this method of simultaneously applied tools “triple stain”. All Gb3 binding molecules were added to fixed cells according to the standard immunofluorescence staining protocol (see Section 4.5). Stained cells were imaged at a confocal fluorescence microscope. Initial results clearly demonstrated that the anti-Gb3 antibody showed a very weak signal in the presence of the other lectins (Figure 3A1,A2), without anti-Gb3 pre-incubation). Using 5 times the molar concentration of the antibody to the nearest lectin (StxB) concentration also did not lead to an increase in antibody binding. On the other hand, the anti-Gb3 antibody showed an appropriate, specific signal when incubated without lectins in control experiments (Supplementary Figure S2). This indicates that the binding of anti-Gb3 was hindered by the presence of lectins.

These findings prompted us to further investigate the Gb3-binding molecules in a “double stain” approach so that the two-color images could be further analyzed using an established pixel-based colocalization method to gain a deeper understanding of the binding kinetics of these molecules. Surprisingly, the anti-Gb3 antibody did not produce a detectable signal when incubated simultaneously with StxB, whereas co-incubation with LecA resulted in a different staining pattern (Supplementary Figure S3). This suggests that StxB is the primary factor, which inhibits the anti-Gb3 antibody from binding to Gb3 in Fabry fibroblasts. To investigate this further, we pre-incubated the anti-Gb3 antibody for one hour before introducing the lectins StxB and LecA along with the secondary antibody against anti-Gb3 (Figure 3B1,B2). This pre-incubation step allowed the anti-Gb3 antibody to bind to Gb3 without competition from lectins. While the anti-Gb3 signal improved after pre-incubation, it remained weak and inconsistent, identifying fewer structures compared to the lectins (Supplementary Figure S4). Thus, we decided to use the double-stain method (with pre-incubation) for a detailed analysis of binding kinetics for different combinations, namely StxB-AF488 vs. anti-Gb3 (AF647), LecA-AF488 vs. anti-Gb3 (AF647), and StxB-AF488 vs. LecA-AF647, using a pixel-based quantification of overlapping signals in Fabry fibroblasts. The colocalization of overlapping signals was quantitatively assessed using the Manders Colocalization Coefficient (MCC): M1 and M2 are providing insights into the complementary binding dynamics of these probes.

**Figure 4:**
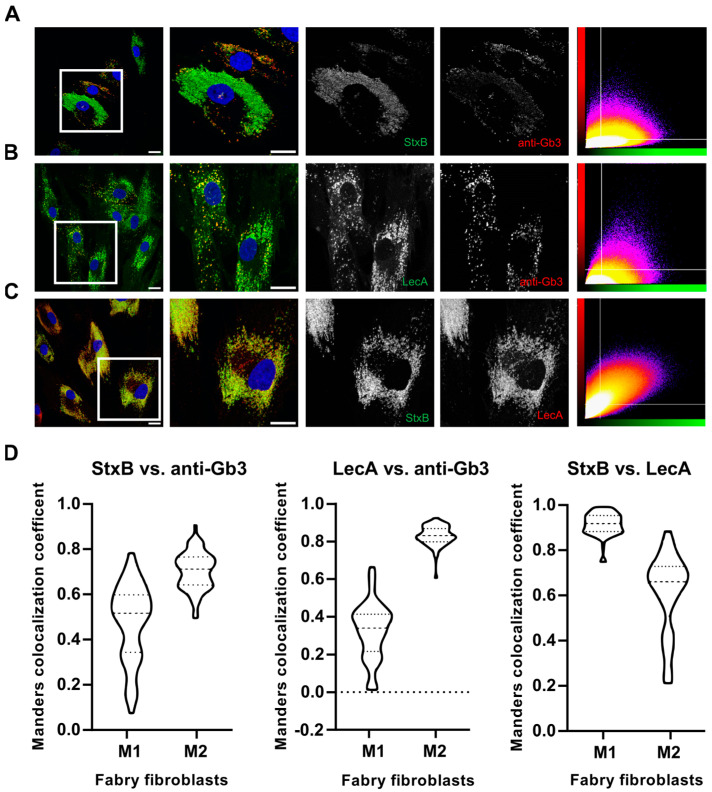
Double stain of Fabry fibroblasts using an anti-Gb3 antibody, StxB, and LecA. Confocal images of Fabry fibroblasts (all panels) depict different staining in the following combinations: StxB-AF488 and anti-Gb3 (anti-rat-AF647) in (**A**), LecA-AF488 and anti-Gb3 (anti-rat-AF647) in (**B**), and StxB-AF488 and LecA-AF647 in (**C**), where anti-Gb3 was pre-incubated in all cases for one hour. Each panel displays a zoomed in area indicated by a white square (1000 × 1000 pixels) of the full field of view (2048 × 2048 pixels), along with grayscale images from individual channels. Scatter plots of the pixel intensity of individual channels are shown for cells from zoomed in areas (far right). White horizontal and vertical lines indicate the automated threshold values determined by ImageJ (2.1.0) using Otsu’s method to segment the region of interest for all images in Fabry fibroblasts. (**D**) The signals for the indicated labels were recorded as Z-stacks for each cell for 3D colocalization analysis using the BIOP-JACoP plugin in ImageJ (*n* ≥ 60 cells). The Manders Colocalization Coefficient (MCC) was used to evaluate the proportion of the fluorescence intensity of one signal that overlaps with the other. M1 represents the proportion of overlapping pixels of the green channel in the red channel, while M2 represents the opposite. MCC values range from 0 to 1.0, where 1.0 stands for perfect colocalization. MCC values are represented using a violin plot, which illustrates the median, interquartile range, outliers, and the probability density of the data. Wider sections of the plot indicate areas with higher data density, while narrower sections represent lower density regions. All scale bars represent 20 µm.

In double stains, StxB and LecA exhibited a broader signal distribution compared to anti-Gb3, even though anti-Gb3 was pre-incubated in double stains in all cases (Figure 4A,B). Additionally, the scatter plots of these conditions (StxB vs. anti-Gb3 and LecA vs. anti-Gb3) indicate poor signal overlap due to the imbalances in signal levels. Points (indicative of each overlapping pixel) are spread across the plot with no clear diagonal pattern, representing that the two channels do not display comparable signal intensities (Figure 4A,B). Conversely, the combination of StxB and LecA shows a high degree of overlap and comparable signal intensities. This is indeed visible in grayscale images of the zoomed in region and in the scatter plot as the pixels form a diagonal line, representative of a strong correlation between pixels with similar intensities (Figure 4C). The MCC analyses depicted in Figure 4D expose that both StxB and LecA (green channel) expose broader signal distribution in double staining compared to the anti-Gb3 antibody stated evidently by the fact that M2 values are higher than M1 values. In the case of StxB vs. anti-Gb3, the M1 and M2 values were 0.48 and 0.70, respectively. For the combination of LecA vs. anti-Gb3, the M1 and M2 values were calculated as 0.33 and 0.84, respectively. Interestingly, for the StxB and LecA combination, M1 was found to be 0.92, indicating a high degree of overlap of StxB with LecA. However, the lower M2 value (0.61) suggests that LecA binds to additional structures that StxB does not. To draw reasonable conclusions with MCC values, we have mimicked the “complete” and “no” overlap scenarios using anti-mouse AF488 and AF647 against anti-Lamp1 antibody on Fabry fibroblasts (Supplementary Figure S5). The BIOP-JACoP plugin was used to calculate M1 and M2 values. Complete overlap was calculated from the Lamp1 signal derived from the Lamp1 signal originating from both AF488 and AF647 (Supplementary Figure S5A); M1 and M2 values were calculated as 0.90 and 0.91, respectively. For the no-overlap case, calculated between the nuclear signal (DAPI) and the signal from AF647 (Supplementary Figure S5B), M1 and M2 values were found to be 0.013 and 0.2, respectively.

Together, these results state that StxB exhibits faster binding kinetics compared to anti-Gb3, and both lectins show more selective binding provided by high avidity. Considered together, their ability to detect not only Gb3 levels but, in some cases, other accumulating substrates associated with Fabry disease underlines their superior utility in this context.

**Figure 5:**
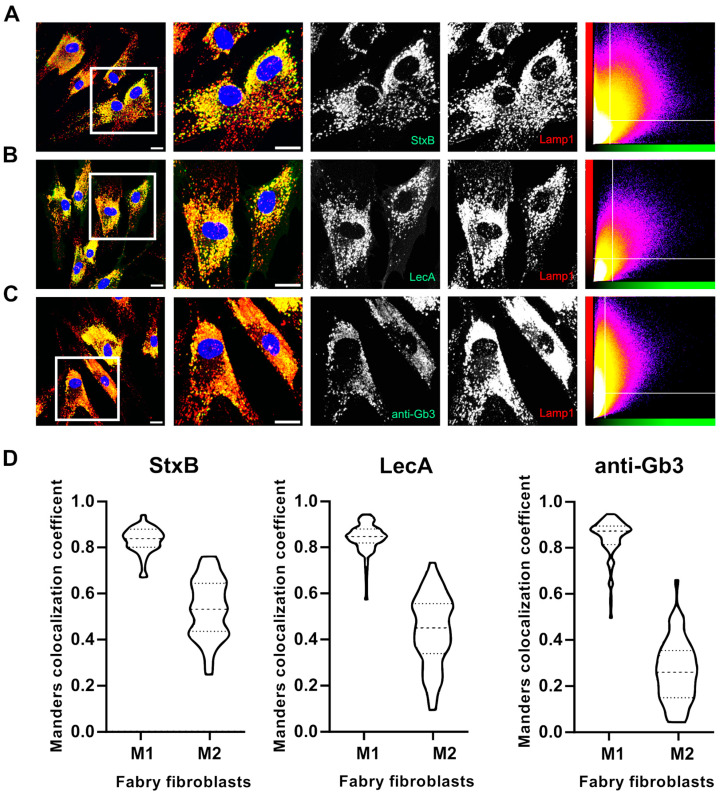
Lysosomal accumulation of Fabry substrates assessed by LecA, StxB, and anti-Gb3. Confocal images of Fabry fibroblasts (all panels), which were stained with an anti-Lamp1 antibody (and a secondary AF647-labeled antibody), are shown. The cells were counterstained with either LecA-AF488 (**A**), StxB-AF488 (**B**), or anti-Gb3 (labeled with anti-rat-AF488 (**C**)). Each panel displays a zoomed in area indicated by a white square (1000 × 1000 pixels) of the full field of view (2048 × 2048 pixels), along with grayscale images from individual channels. Scatter plots of the pixel intensity of individual channels are shown for cells from zoomed in areas (far right). White horizontal and vertical lines indicate the automated threshold values determined by ImageJ using Otsu’s method to segment the region of interest for all images in Fabry fibroblasts. (**D**) The signals for the indicated labels were recorded as Z-stacks for each cell for 3D colocalization analysis using the BIOP-JACoP plugin in ImageJ (*n* ≥ 60 cells). The Manders Colocalization Coefficient (MCC) was used to evaluate the proportion of the fluorescence intensity of one signal that overlaps with the other. M1 represents the proportion of pixels of the green channel that overlap with the red channel, while M2 represents the opposite. MCC values range from 0 to 1.0, where 1.0 stands for perfect colocalization. MCC values are represented using a violin plot, which illustrates the median, interquartile range, outliers, and the probability density of the data. Wider sections of the plot indicate areas with higher data density, while narrower sections represent lower density regions. All scale bars represent 20 µm.

In a next step, we used these three molecules to investigate the lysosomal accumulation of Gb3 in Fabry fibroblasts. StxB, LecA, and an anti-Gb3 antibody were used individually to label the accumulating Fabry substrates along with an anti-Lamp1 antibody to detect lysosomes. Fabry fibroblasts exhibited a pronounced lysosomal content, both in terms of abundance and overall morphology, compared to their healthy counterparts (Supplementary Figure S6). The LecA signal shows greater signal overlay with anti-Lamp1 (Figure 5B) compared to StxB (Figure 5A) and anti-Gb3 (Figure 5C), which is evident in the grayscale images and the scatter plots in Figure 5. M1 was calculated to be 0.83 for both LecA and StxB. On the other hand, anti-Gb3 revealed a slightly higher M1 value of 0.84. In contrast, M2 values showed a remarkable decrease with values of 0.53, 0.43, and 0.26 for LecA, StxB, and anti-Gb3, respectively. The same trend was also observed in healthy fibroblasts (Supplementary Figure S7). These results reaffirm that the signal from all probes correlates with lysosomes presented by M1 values. Hence, M2 values clearly show that the proportion of the anti-Gb3 signal is lower in lysosomes compared to StxB or LecA. Notably, LecA emerges as the most effective lectin for providing insights into the lysosomal accumulation of Fabry substrates. Additionally, StxB, as a highly specific marker for Gb3, demonstrates superior representation of lysosomal accumulation compared to its closest alternative, the anti-Gb3 antibody.

### 2.4. The Sphingolipid Metabolism Is Disturbed in Fabry Cells as Revealed by Mass Spectrometry

**Figure 6:**
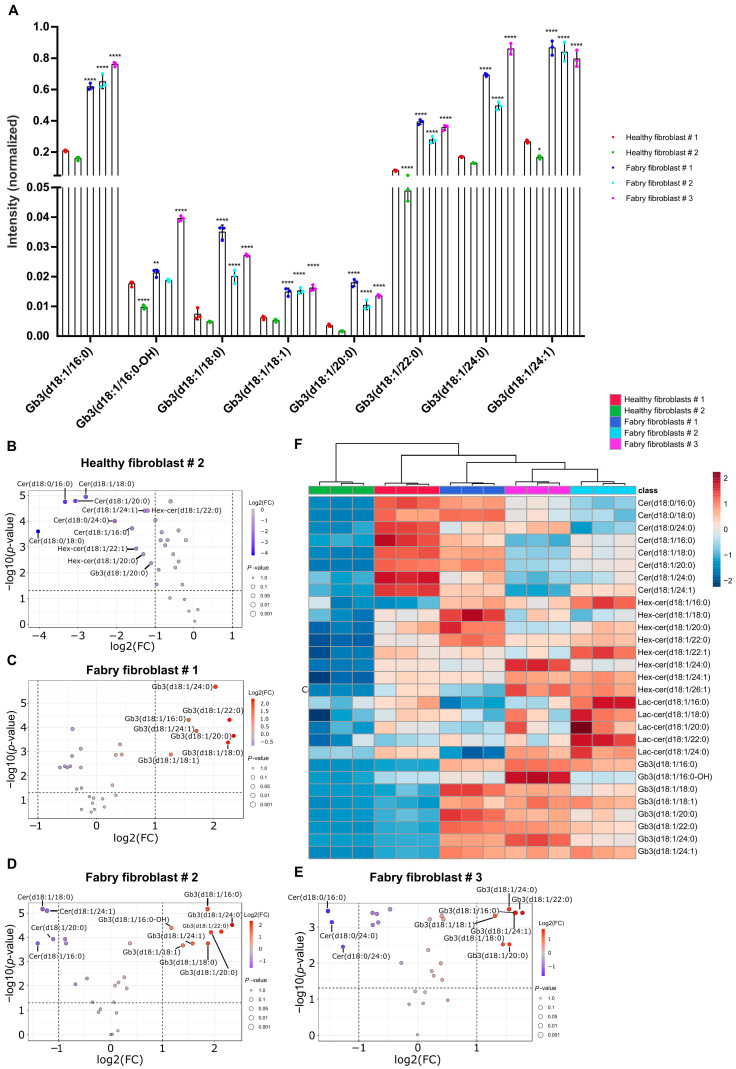
Patient-derived Fabry fibroblasts display an enrichment of Gb3. Three Fabry fibroblast cell types (which bear different mutations and display the classical phenotype) and two healthy fibroblast cell types (from different donors who were 3 (HF2) and 38 years (HF1) old) were used. (**A**) Intensity peaks indicating the amount of Gb3 isoforms in Fabry and healthy fibroblasts were measured by targeted LC/MS analysis. Data are means; error bars are standard deviation (SD). The significance of the differences tested by one-way ANOVA was followed by Dunnett’s multiple comparisons test (*n* = 3). Error bars are means ± (SD). Only significant values are displayed. * *p* < 0.05, ** *p* < 0.01, **** *p* < 0.0001. (**B**–**E**) Volcano plots are showing log2 fold changes (FC) on the *x*-axis and negative decadic logarithm of *p*-values after false discovery rate-based multiple testing correction on the ordinates (dashed lines represent cut-offs of 0.5 and 2 for fold change and q < 0.05). Increased values are depicted as red, reduced values as blue. Only lipids matching both cut-offs were labeled. (**B**) HF2 vs. HF1, (**C**) FF1 vs. HF1, (**D**) FF2 vs. HF1, (**E**) FF3 vs. HF1. (**F**) Heat map analysis of significantly changed lipids according to one-way ANOVA and false discovery rate-based multiple testing correction (q < 0.05). Range-scaled z-scores are displayed. The results are presented in technical triplicates and have been reproduced in biological replicates.

Due to the impaired activity of α-Gal A, Fabry cells display abnormalities in Gb3 metabolism that may extend beyond the accumulation of Gb3 and lyso-Gb3. While these metabolites have been the primary focus of many studies utilizing animal models and analyzing plasma and urine samples from Fabry patients, possible broader metabolic implications designate a need for further investigation [3,40,41]. One factor that impacts the Gb3 accumulation is the sphingolipid (SL) metabolism of the cell [42]. In this study, we have aimed to address the impaired SL profile in mammalian cultured cells. A comprehensive comparison of fibroblasts from two healthy individuals with age differences and three Fabry patients was performed to clarify the aberrant SL metabolism in three different *GLA* mutations and to gain a first insight into the correlation between genotype and phenotype. Furthermore, conditionally immortalized human podocytes from both *GLA*-knockout (KO) and wild-type (WT) models were analyzed under the same conditions. Our primary objective was to identify sphingolipid species, focusing on fatty acyl chain length, degree of saturation, and hydroxylation. Briefly, cell lysates were prepared by homogenizing cells in a 1:1 methanol-to-water mixture, followed by chloroform-based lipid extraction [43]. The resulting lipids were subjected to liquid chromatography and targeted mass spectrometry for detailed analysis. Normalized intensity values were used to quantify the total abundance of Gb3 isoforms. From now on, the Fabry fibroblast cell lines will be referred to as FF1, FF2, and FF3, while the healthy fibroblast lines will be referred to as HF1 and HF2. It is important to note that HF1 and FF1 were the cell types used previously in flow cytometry and immunofluorescence experiments. Figure 6A illustrates the relative abundance of various sphingolipid species in Fabry and healthy fibroblasts. In Fabry fibroblasts (FF1, FF2, and FF3), certain Gb3 isoforms exhibited relatively high abundance compared to total abundance of all targeted Gb3-isoforms, including C16:0 (23%, 26%, and 26%), C22:0 (15%, 12%, and 12%), C24:0 (26%, 25%, and 30%), and C24:1 (33%, 33%, and 27%). In contrast, other Gb3 isoforms were present at significantly lower levels, such as C16:0-OH (0.79%, 1%, and 1.4%), C18:0 (1.3%, 0.93%, and 0.94%), C18:1 (0.5%, 0.6%, and 0.6%), and C20:0 (0.7%, 0.5%, and 0.47%). A similar trend was observed in healthy fibroblast HF1, where Gb3 isoforms such as C16:0 (27%), C22:0 (11%), C24:0 (22%), and C24:1 (35%) exhibited relatively high abundance, while other isoforms were detected at markedly lower levels.

To summarize, all Gb3 isoforms were significantly increased in Fabry fibroblasts (FF1, FF2, FF3) compared to healthy fibroblasts HF1 (*p* < 0.0001), except for C16:0-OH, which showed no significant difference in FF2. No significant differences regarding Gb3 isoforms were observed between the two healthy fibroblast lines (HF1 and HF2). The volcano plots in Figure 6B–E illustrate the upregulated and downregulated glycosphingolipids in various comparisons. In particular, these plots compare HF1 with HF2 (Figure 6B), FF1 (Figure 6C), FF2 (Figure 6D), and FF3 (Figure 6E). The intensity peaks and statistical significance values of other targeted Gb3 precursors, including ceramides (Cer), hexosyl-(HexCer), and lactosylceramides (LacCer), are provided in the Supplementary Information (Supplementary Figure S8). Both Hex- and LacCers displayed an increasing tendency in the comparison between HF1 and HF2 (Figure 6B), which reflects differences between the older donor (HF1) and the younger donor (HF2). Interestingly, ceramides showed a significant increase in all isoforms in HF1 compared to HF2, as well as compared to all other Fabry fibroblasts. The heat map in Figure 6F further confirms this upregulation. All ceramides showed a significant increase in HF1 when compared with Fabry fibroblasts (FF1, FF2, FF3), with the exception of Cer(d18:1/18:0) in FF1. However, hexosyl- and lactosylceramides did not follow a consistent trend and showed no significant differences between the Fabry fibroblasts, as can be clearly seen in both the volcano plots and the heat map (Figure 6). These results emphasize the different Gb3 profiles between Fabry fibroblasts (FF1, FF2, FF3) and healthy fibroblasts (HF1, HF2) and highlight the significant differences in the distribution of Gb3 isoforms in the different cell types.

**Figure 7:**
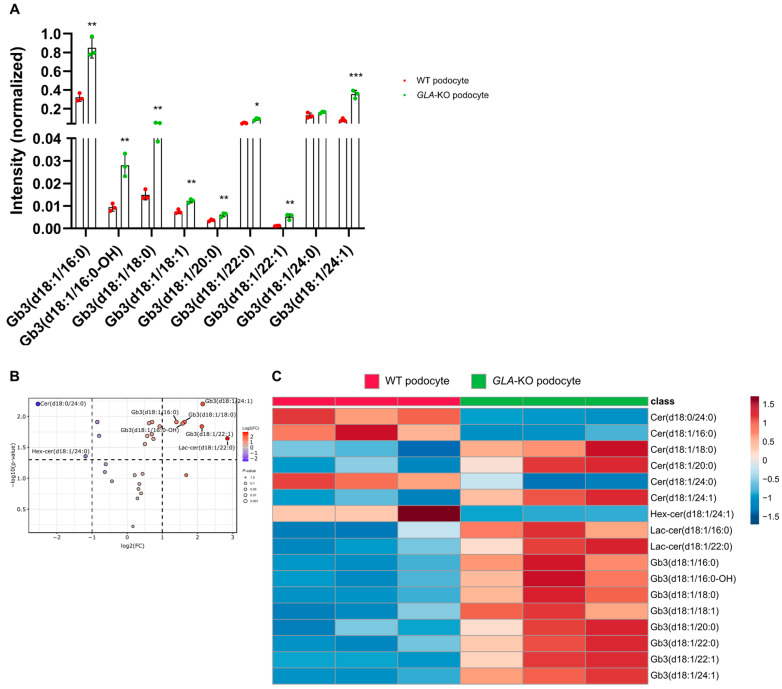
*GLA*-KO Fabry podocytes display upregulated Gb3 metabolism. (**A**) Intensity peaks displaying the quantity of Gb3 isoforms in *GLA*-KO and WT podocytes were determined by targeted LC/MS analysis. Error bars are means ± (SD). Significance of the differences tested by unpaired two-tailed *t*-test. Only significant values are displayed. * *p* < 0.05, ** *p* < 0.01, *** *p* < 0.001. (**B**) Volcano plot shows log2 fold changes (FC) on the *x*-axis and negative decadic logarithm of *p*-values after false discovery rate-based multiple testing correction on the ordinate (dashed lines represent cut-offs of 0.5 and 2 for fold change and q < 0.05). Increased values are depicted as red, reduced values as blue. Only lipids matching both cut-offs were labeled. (**C**) Heat map analysis of significantly changed lipids according to *t*-test analysis and false discovery rate-based multiple testing correction (q < 0.05). Range-scaled z-scores are displayed. The results are presented in technical triplicates and have been reproduced in biological replicates.

Additionally, we performed the same experimental approach with *GLA*-knockout (KO) and WT podocytes. The results closely resembled those observed in primary fibroblasts, with all Gb3 isoforms upregulated in *GLA*-KO podocytes compared to WT podocytes (Figure 7). In *GLA*-KO podocytes, certain Gb3 isoforms exhibited relatively high abundance, including C16:0 (55%), C22:0 (5.8%), C24:0 (10%), and C24:1 (22%). In contrast, other Gb3 isoforms were present at significantly lower levels, such as C16:0-OH (1.8%), C18:0 (3.0%), C18:1 (0.8%), C20:0 (0.4%), and C22:1 (0.3%). A similar tendency was observed in WT podocytes, where Gb3 isoforms such as C16:0 (52%), C22:0 (7.7%), C24:0 (21%), and C24:1 (13%) exhibited relatively high abundance, while other isoforms were detected at markedly lower levels, including C16:0-OH (1.5%), C18:0 (2.4%), C18:1 (1.2%), C20:0 (0.6%), and C22:1 (0.3%). All Gb3 isoforms showed significantly increased abundances in *GLA*-KO podocytes compared to WT podocytes, except for the C24:0 isoform. In particular, Gb3-C24:0 was the only isoform not statistically significant, while the remaining Gb3 isoforms exhibited differences statistically significant.

## 3. Discussion

Lectins have emerged as excellent tools for the detection of the glycosphingolipid Gb3 and have proven their superiority over anti-Gb3 antibodies as markers in glycan detection assays, as recent developments show. Lectin-based glycan arrays are increasingly recognized in glycan research for their high specificity and lower susceptibility to cross-reactivity often seen with commercial antibodies [19,21,22,33]. Notably, commercially available antibodies have been criticized in protein research because their efficiency in detecting glycolipids has been questioned in comparison to the natural glycan recognition capabilities of lectins [44]. In this study, the two Gb3-binding lectins, StxB and LecA, were used as tools for the detection of glycans. Schubert et al. applied these lectins simultaneously to stain the plasma membrane of Gb3-positive MDCK cells. Confocal microscopy revealed intriguing findings, namely that StxB and LecA were segregated into distinct domains at the plasma membrane of cells, leading to further analysis of the binding preferences of these lectins, using giant unilamellar vesicles (GUVs) as a membrane model system. They discovered that both lectins bind to Gb3 in an isoform-specific manner influenced by the Gb3 fatty acyl chain structure and the local membrane environment, specifically liquid-ordered and liquid-disordered domains. The results demonstrated that StxB preferentially binds to the C18:1 and C24:0 isoforms of Gb3 in liquid-ordered domains, whereas LecA exhibits a distinct binding pattern by binding Gb3 species with shorter fatty acid chains and showing a higher affinity for liquid-disordered membrane domains. Moreover, StxB recognized Gb3 at the primary cilium and the periciliary membrane, whereas LecA only bound periciliary Gb3 in polarized MDCK cells [28]. Other studies have engineered and utilized bispecific lectin chimeras termed “lectibodies” in innovative therapeutic approaches. StxB was genetically fused to an anti-CD3 single-chain antibody fragment to target and kill Gb3-positive cancer cells by activating cytotoxic T cells. This approach represents a promising strategy for improving glycan target specificity against cancer cells [45]. Furthermore, lectin-based CAR-T cells (also called lectin-CAR T cells) have been developed by conjugating the sequences of a second-generation CAR and a Gb3-binding lectin (StxB, LecA, and Mitsuba) as antigen-binding domains, which target and kill various cancer cells [39]. These advancements highlight the growing versatility of lectins in glycan recognition and therapeutic applications.

In Fabry disease, the combination of StxB and LecA offers significant advantages, not only in the detection of Gb3 accumulation but also in the identification of other α-D glycol-conjugates, which are the primary molecular structures that accumulate in the disease [2]. Although Gb3 is the main substrate accumulating in Fabry disease, other α1,3- and α1,4-linked galactosyl residues, such as globotriaosylsphingosine (lyso-Gb3, the deacylated form of Gb3), digalactosylceramide (Gal2Cer), B antigen, and P1 antigen, also accumulate to a lesser extent in lysosomes [4]. Previously, StxB and Isolectin B4 (from *Griffonia simplicifolia*) have been used individually in Fabry research, including immunofluorescence and immunohistochemistry, to target Gb3 and α-D glycol-conjugates [46,47,48]. However, we strongly doubt that these tools used individually can adequately capture all Gb3 isoforms and analogs that accumulate in Fabry cells, such as lyso-Gb3, as there are structural differences that could alter the presentation of the Gb3 head group. StxB can successfully target certain Gb3 species but may not comprehensively recognize all Fabry substrates. Therefore, the inclusion of LecA as an additional Gb3-binding lectin is critical for a complete assessment of substrate accumulation in Fabry cells [28]. One other importance of LecA in FD is a study published in 2018 clearly demonstrating that LecA reveals hetero-multivalent interaction by simultaneously binding Gb3 and LacCer, efficiently increasing its binding to Gb3 in model membranes [49]. It is important to note that LacCer is not a direct substrate accumulating in FD, but due to the disturbed Gb3 degradation, it can be assumed that it also accumulates due to the far-reaching disturbances in the processing of glycosphingolipids. However, our mass spectrometry results demonstrate that there is no particular trend in LacCer species in either Fabry fibroblasts or podocytes. Nonetheless, this potential impairment of LacCer abundance could have a positive impact on the avidity of LecA for Gb3, further emphasizing its utility in detecting glycosphingolipid imbalances in Fabry disease.

The colocalization analysis performed in this study using Manders Colocalization Coefficients supports the complementary use of these two lectins. A high overlap (M1 = 0.92) between StxB and LecA signals, combined with a lower M2 value (0.62) for LecA, suggests that LecA can provide unique and additional information about Fabry fibroblasts. In comparison, colocalization of anti-Gb3 and StxB resulted in a low M1 value (0.48), supporting the hypothesis that anti-Gb3 antibodies are less effective at accurately binding Gb3 compared to StxB. However, the calculated M2 value (0.70), which is closer to our control M2 value (0.90), still indicates that not all anti-Gb3 signals overlap with StxB. For LecA and anti-Gb3, the M1 and M2 values were calculated to be 0.33 and 0.84, respectively, clearly indicating that when comparing both lectins against anti-Gb3, LecA provides more comprehensive coverage than StxB (Figure 4). These findings strongly suggest that both sequential and simultaneous use of StxB and LecA improves the detection of substrate localization in Fabry cells and highlights the advantages of lectins in glycan detection during immunofluorescence staining [47].

Furthermore, a comparison of all Gb3-binding molecules in the lysosomal accumulation revealed that LecA provides the most comprehensive information about substrate accumulation in lysosomes in Fabry fibroblasts. Notably, LecA exhibited the highest M2 value (0.53), indicating that it achieves the highest overlap with the Lamp1 and is very effective in identifying Gb3 accumulation within lysosomes. This highlights the superior ability of LecA to recognize and map glycosphingolipid perturbations in Fabry cells, while StxB (0.43) accurately maps the Gb3 population, performing much better than the anti-Gb3 antibody (0.26).

The relatively low M2 values calculated for Gb3-binding molecules against Lamp1 staining (the experimental perfect colocalization M2 value of 0.90, Supplementary Figure S5) may be attributed to the presence of lysosomal structures that are not fully occupied by glycoconjugates and not to unbalanced signal intensities between Lamp1 and Gb3-binding molecules. This issue was addressed by Manders et al., who advocated the use of MCC as a more robust measure to resolve these discrepancies as opposed to Pearson correlation coefficient [47,48].

Also in flow cytometry experiments, commercially available anti-Gb3 antibodies demonstrated a disappointing performance (Figure 1). To our surprise, these antibodies failed to bind Gb3 in flow cytometry of H1299 cells, Fabry, and healthy fibroblasts. This failure can only be explained by the fact that antibodies are cell-type specific. One can argue about the possibility that an anti-Gb3 antibody cannot recognize Gb3 due to steric hindrance by other membrane components in the plasma membrane [23,24]. Indeed, Fabry fibroblasts and H1299 cells were also subjected to whole-cell flow cytometry analysis, in which they were fixed and permeabilized prior to antibody incubation. This approach effectively rules out the hypothesis of a steric hindrance for fibroblasts, as no significant increase in fluorescence intensity was observed compared to untreated cells. Interestingly, H1299 cells showed a slight increase in fluorescence intensity, which could be due to alterations in the plasma membrane caused by PFA fixation or improved accessibility to intracellular Gb3 deposits due to permeabilization. Nonetheless, these results once again highlight the inconsistency of antibody performance across cell types and preparation methods. We are confident that the inconsistency of the anti-Gb3 antibody is not related to Gb3 levels or isoform abundance, as both Ramos and H1229 carcinoma cell lines showed similar upregulated Gb3 metabolism, which was assessed by whole-cell MS analysis, revealing a similar trend for Gb3 isoforms [35,39]. In contrast, lectins have proven their superiority by providing important insights from plasma membrane and whole-cell analyses in flow cytometry experiments, emphasizing their versatility and broader applicability in detecting Gb3 in different cell types (Figure 2).

The accumulation of substrates resulting from impaired SL metabolism varies considerably between cell types affected by FD, depending on their specific sphingolipid metabolic pathways [42]. The results of gene therapy with lentiviruses in Fabry patients may indirectly confirm these discrepancies in GSL metabolism between individuals [50]. This variability could also be an explanation for the discrepancies observed in Fabry mouse models [40,50,51]. On the other hand, human studies have mainly focused on analyzing body fluids from Fabry patients using mass spectroscopy. Hence, it was very important to use cultured human cells, including primary and knockout (KO) models, to fill this knowledge gap, investigate substrate accumulation from different angles, and improve our understanding of GSL accumulation in Fabry disease. We used primary fibroblasts from three different donors that showed the classical phenotype with a residual α-Gal A activity of up to 27% [52]. In addition, we also used human *GLA*-KO podocytes, as this is the most affected cell type in the human glomerulus in Fabry disease [53]. Although cultured human cells have not been the focus of MS studies for substrate accumulation profiling, one notable study used cultured patient-derived fibroblasts to assess the reduction of Gb3, lyso-Gb3 isoforms, and other SLs following GSL inhibition by lucerastat [54,55,56]. We additionally used two healthy fibroblast cells from 38- and 3-year-old donors to investigate a possible age-related change in SL levels. Our results showed that the measured Gb3 isoforms were upregulated in Fabry fibroblasts, with the exception of C16-OH in FF2. An increasing trend is also observed in ceramide analogs (d18:0) and isoforms (d18:1) in HF1 compared to HF2, but no significant differences are observed among Gb3 isoforms (Figure 6A,B,F). This suggests that age might influence SL metabolism but is not necessarily a factor influencing Gb3 levels. All Gb3 isoforms were significantly increased in all Fabry cells (with the exception of C16-OH in FF2, Figure 6A,C,D,F). In podocytes, C24 was the only isoform that did not increase significantly, although KO mice had longer fatty acyl chain isoforms in kidney tissues [40]. The abundances of Gb3 isoforms in podocytes correlated with the study performed on the same cell line [57] (Figure 7). Other GSLs, including the Hex- and LacCer isoforms, showed relatively increased levels in Fabry fibroblasts, although there was no particular trend between the different isoforms (Supplementary Figure S8). Hex- and LacCer isoforms also did not show a trend consistent with donor-derived cells and the KO Fabry model (Supplementary Figure S9). In summary, all Gb3 isoforms were increased in both cell lines, with the Gb3 precursor GSLs following an upward or downward trend that varied between the different donors, and the same trend was also present in the *GLA*-KO model, which could be a first explanation for the discrepancies in SL studies in Fabry research.

To conclude, lectins have great potential as reliable detection tools for glycolipids, such as Gb3. Their fast binding kinetics are particularly advantageous, as evidenced by the absence of anti-Gb3 signals in the presence of StxB. Moreover, the combined use of StxB and LecA enables a more comprehensive understanding of the accumulating substrates in Fabry disease and thus better detection and characterization. In particular, given the aberrant GSL metabolism and the diversity of accumulating substrates in Fabry cells, it is important to address glycan accumulation with alternative lectins. Since we were able to detect different Gb3 concentrations using lectins, ranging from low (in healthy cells) to high (in several Fabry fibroblast cells with differences in the residual α-Gal activity), we are convinced that it is also possible to quantify Gb3 concentrations after therapeutic interventions. Finally, yet importantly, the ability to produce these lectins in the laboratory ensures a scalable and accessible approach for future research and diagnostic applications.

## 4. Materials and Methods

### 4.1. Antibodies and Chemicals

The following anti-Gb3 antibodies were used in flow cytometry experiments: anti-CD77-FITC (anti-Gb3 # 1) derived from rat (Antibodies.com, St. Louis, MO, USA, Cat. No. A285834), anti-CD77-FITC (anti-Gb3 # 2) derived from mouse (BD Pharmingen^TM^, Franklin Lakes, NJ, USA, Cat. No. 551353), and anti-CD77-FITC (anti-Gb3 # 3) derived from mouse (BioLegend, San Diego, CA, USA, Cat. No. 357103). The following primary antibodies were used in immunofluorescence experiments: anti-CD77 derived from rat (Antibodies.com, St. Louis, MO, USA, Cat. No. A254401) and anti-CD107a (Lamp1) derived from mouse (BD Pharmingen^TM^, Franklin Lakes, NJ, USA, Cat. No. 611042I). The following secondary antibodies were used: anti-rat IgG-Alexa Fluor 488-conjugated (Thermo Fisher Scientific, Waltham, MA, USA, Cat. No. A21208), anti-rat IgG-Alexa Fluor 647-conjugated (Thermo Fisher Scientific, Waltham, MA, USA, Cat. No. A-31573), and anti-mouse IgG-Alexa Fluor 647-conjugated (Thermo Fisher Scientific, Waltham, MA, USA, Cat. No. A21236).

The following chemicals and solutions were used for the cell culture and experiments: DPBS 1X, without CaCl_2_ and MgCl_2_ (Cat. No. AC-BS-0002), Penicillin-Streptomycin Solution 100X (Cat. No. AC-AB-0024), RPMI 1640 1X (Cat. No. AC-LM-0060), and Trypsin-EDTA 1X (Cat. No. AC-EZ-0004) were purchased from Anprotec, Bruckberg, Germany. L-Glutamine 200 mM (Cat. No. 7-GLN-B) was ordered from 7Bioscience GmbH, Neuenburg am Rhein, Germany. DMEM 1X (Cat. No. 21969-035) and fetal calf serum (FCS, Cat. No. 10270106) were purchased from Gibco by Thermo Fisher Scientific, Waltham, MA, USA. PE Annexin V (Cat. No. 556454) was obtained from Becton, Dickinson, and Co (BD Biosciences), San Jose, CA, USA, NH_4_Cl (Cat. No. K298.1), EDTA (Cat. No. 8043.1), paraformaldehyde (PFA, Cat. No. 335.1), DMSO (Cat. No. A994.2), and DAPI (Cat. No. 4855.2) were purchased from Carl Roth GmbH and Co. KG., Karlsruhe, Germany, Alexa Fluorophore 488 NHS Ester (Cat. No. Thermo Fisher Scientific obtained A20000) from Invitrogen, Carlsbad, CA, USA. DABCO solution (Cat. No. D27802), StxB (Cat. No. SML0562-0.5MG), Mowiol (Cat. No. 81381-250G), saponin (Cat. No. 84510-100G), and insulin-transferrin-sodium selenite media supplement (ITS, Cat. No. 11074547001) were ordered from Sigma-Aldrich Inc., Darmstadt, Germany.

### 4.2. Cell Lines

Primary cultures of fibroblasts derived from Fabry disease patients with mutations in their *GLA* gene were used alongside fibroblasts from healthy individuals. Fabry fibroblast # 1 carries the c.639+5G>T mutation, while Fabry fibroblast # 2 carries the c.680G>A mutation with a very low remaining α-Gal activity of approximately 5 nmol/h/mL [58]. Fabry fibroblast # 3 carries the c.485G>A mutation reported in two different studies with an α-Gal activity ranging from 10–15% and 27%, respectively, of a normal enzymatic activity [52]. FF1, FF2, and FF3 were 41-year-old, 38-year-old, and 10-year-old male patients at sampling, respectively. Healthy fibroblast #1 and healthy fibroblast #2 were derived from 38-year-old and 3-year-old male individuals, respectively [59].

Conditionally immortalized podocytes were developed to enable proliferation at permissive temperatures (33 °C) and differentiation at non-permissive temperatures (37 °C) by transfecting the temperature-sensitive SV40-T gene [60]. Fabry podocytes (*GLA*-KO) were generated by Christoph Schell and Tobias Huber. The *GLA* gene was knocked out in conditionally immortalized podocytes using CRISPR-Cas9 technology [10].

### 4.3. Cell Culture

RPMI 1640 medium supplemented with L-glutamine, 10% fetal calf serum (FCS), 1% penicillin/streptomycin, and 5 mg ITS (aliquoted in H_2_O) was used. Additional supplements included HEPES (1 M, 1:200), MEM non-essential amino acids (NEAA, 100×, 1:1000), and sodium pyruvate (100 mM, 1:1000). Cells were sub-cultured in 10 cm culture dishes at a 1:5 ratio until they reached 80–90% confluency at 33 °C. For differentiation, 1 million cells were seeded in a 10 cm dish and cultured overnight at 33 °C and 5% CO_2_. On the next day, podocytes were transferred to 37 °C and 5% CO_2_ for 12–14 days, with media changes every 3 days.

Primary cultures of human fibroblasts were grown in DMEM supplemented with 4 mM L-glutamine, 1% penicillin-streptomycin solution (100×), and 10% fetal calf serum (FCS). Fibroblasts were cultured at 37 °C and 5% CO_2_ until 80–100% confluence and then split into T25 flasks at a 1:2 ratio. The cells were used up to a maximum passage number of 30.

### 4.4. Lectin Labelling

LecA and StxB were labeled with fluorescent dyes for flow cytometry and confocal microscopy. The labeling was performed through NHS-ester amine conjugation, using Alexa Fluor 488, 555, and 647 (AF488, AF555, and AF647). The respective lectin was dissolved in ddH_2_O and supplemented with 10% (*v*/*v*) 1 M NaHCO_3_ at pH 8.5. The dye solution was dissolved in DMSO at a concentration of 10 mg/mL and was added to the lectin solution. The volume of the dye solution to be added was calculated using the following equation, $V_{d}=\frac{C_{p}\times V_{p}}{{MW}_{p}}\times ME\times \frac{{MW}_{d}}{C_{d}}$ , where c_p_ = protein concentration, V_p_ = volume of protein label, MW_p_ = the molecular weight of the protein, ME = molecular excess (the number of dye molecules per protein molecule), V_d_ = volume of the dye solution to be added, and c_d_ = concentration of the marker.

The protein and the dye solutions were incubated for one hour at room temperature and were stirred every 5 min. A ZebaTM Spin Desalting Column was prepared by removal of the storage solution by centrifuging the column at 1500× *g* for one minute and then washing it three times with 300 µL of 1 M NaHCO_3_ at 1500× *g* for one minute. The lectin solution was loaded into the column and placed in a collective tube. The column was centrifuged at 1500× *g* for two minutes, and the sample was collected. In the end, the concentration of the labeled protein was measured using NanoDrop (Thermo Fisher Scientific, Waltham, MA, USA).

### 4.5. Immunofluorescence Staining

30,000–40,000 fibroblasts per well were seeded in 4-well plates with 12 mm glass coverslips in 500 μL of fibroblast medium and incubated overnight at 37 °C. The cells were washed with PBS, fixed with 250 μL paraformaldehyde (PFA) 4% in PBS for 15 min, and washed twice with PBS. The PFA was quenched using 250 μL of NH_4_Cl 50 mM for 10 min, followed by two washes with PBS. The cells were blocked and permeabilized for 30 min using 500 μL Sapo solution (0.2% saponin and 3% BSA in PBS). The above-mentioned steps were carried out at RT. The coverslips with the fixed cells were then placed for a one-hour incubation in the dark on 50 μL drops of primary antibodies against the molecule of interest or drops of labeled lectins that were deposited on parafilm. The antibodies were diluted 1:100 or 1:200, and the lectins were diluted to 10 μg/mL in the Sapo solution. The cells were washed three times with PBS, and the coverslips were placed on 50 μL drops of diluted secondary antibodies conjugated to fluorophores (1:200 dilution) for 30 min of incubation in the dark. The cells were washed once with PBS and placed for 10 min incubation in the dark on 50 μL drops of diluted DAPI for nuclear staining (1:500 dilution). The cells were washed twice with PBS, rinsed with ddH_2_O, and mounted on 8 μL drops of mowiol containing DABCO on glass slides. The slides were dried overnight at room temperature in the dark and then stored at 4 °C. The samples were imaged not longer than two days after the staining process.

### 4.6. Image Acquisition

The samples were imaged using a Nikon A1R confocal laser scanning system with a 12-bit intensity range. The setup included a Nikon Eclipse Ti-E inverted microscope with a 60× oil immersion objective with a numerical aperture of 1.49. Excitation of DAPI and Alexa Fluorophores (AF) 488, 555, and 647 was achieved using laser lines at 404.7 nm, 489.0 nm, 562.3 nm, and 641.8 nm. Emission was recorded with filters set at 425–475 nm, 500–530 nm, 570–620 nm, and 663–738 nm, respectively. The pinhole diameter was adjusted to 1.2 A.U. for the 647 nm channel (46.0 µm). The pixel size was established at 0.1 × 0.1 µm for an optimal sampling rate according to the Nyquist theorem. The acquisition parameters (laser intensity, detector gain, and offset values) were optimized for an optimal signal gain to prevent signal oversaturation and fluorophore bleaching. Once the settings were optimized, acquisition parameters kept the same across all samples within a replicate to enable judgments of signal intensity between Fabry and healthy fibroblasts. Special care was taken to avoid oversaturation whenever it was possible. Image volumes were obtained by collecting a series of vertical images (Z stack) with a 0.175 µm step size. Image acquisitions were performed using the NIS-Elements software (version 4.5, Nikon Instruments Europe B.V., Amsterdam, The Netherlands).

### 4.7. Quantitative Colocalization Analysis

Manders Colocalization Coefficients (MCC), M1 and M2, were employed to quantitatively analyze the overlap between signals in dual-color confocal microscopy images. M1 represents the proportion of signal from marker X that overlaps with regions containing signal from marker Y, while M2 indicates the proportion of signal from marker Y that overlaps with regions containing signal from marker X. MCC values range from 0 (no overlap) to 1 (complete overlap) [61]. To exclude background noise and focus on marker-specific signals, Otsu’s thresholding method [62] was applied to all images of Fabry fibroblasts. Overlap values and intensity scatter plots were calculated with the FIJI ImageJ 2.1.0 software using the plugin BIOP-JACoP (Just Another Colocalization Plugin) for all Z-stacks of the cells [63]. The cell recorded cell volume. Cell boundaries were delineated manually using ImageJ’s freehand tool [64].

### 4.8. Flow Cytometry

A suspension of 100,000 cells per well was prepared in a U-bottom 96-well plate. The cells were pelleted by centrifugation at 1600× *g* at 4 °C for 3 min, and the supernatant was discarded. To initiate lectin stimulation, 100 μL of lectin solution in fibroblast medium (at the desired concentration) was added to each well, and the pellet was resuspended in the solution. In the case of anti-Gb3 incubation, 3 µL of antibody solution was diluted in 100 µL FACS buffer (3% BSA in PBS). The cells were incubated at 4 °C for 30 min, protected from light, followed by centrifugation at 1600× *g* at 4 °C for 3 min. The pellets were resuspended with 100 μL of Annexin-V binding buffer (1×) from BD Pharmingen^TM^, Franklin Lakes, NJ, USA. In samples tested for apoptosis, 5 μL of PE Annexin V was added. If apoptosis was not tested, the cells were resuspended in 500 μL FACS buffer after two washes. The samples were then incubated for 15 min in the dark at room temperature, transferred to 1.5 mL tubes containing 400 µL Annexin-V binding buffer, and analyzed within 30 min. The fluorescence intensities of the samples were measured immediately using the Attune NxT flow cytometer (Thermo Fisher). The data were then analyzed using FlowJo V.10 and GraphPad Prism8 software. In flow cytometry experiments with whole cells, the same amount of fibroblasts was fixed with 250 μL paraformaldehyde (PFA) 4% in PBS for 15 min and washed twice with PBS. The PFA was quenched with 250 μL of NH_4_Cl (50 mM) for 10 min, followed by two washes with PBS in a 96-well plate with U-bottom. The cells were blocked and permeabilized for 30 min using 500 μL Sapo solution (0.2% saponin and 3% BSA in PBS). Lectin incubation was then performed for 1 h at RT and then analyzed using the Attune flow cytometer.

### 4.9. Liquid Chromatography-Mass Spectrometry (LC/MS) Analysis of Glycosphingolipids

Fibroblasts (5 × 10^5^) were seeded in 35 mm wells as triplicates the day before the sample preparation. 1 × 10^5^ podocytes were seeded in 35 mm wells as triplicates and incubated overnight at 33 °C. The next day, cells were transferred to 37 °C for differentiation for 12–14 days.

Glycosphingolipids were measured by the method adapted from Lagies et al. [43]. Briefly, cells were washed with 0.9% NaCl (in MilliQ-water), lysed with 1 mL ice-cold methanol/water (1:1 *v*/*v*), scrapped off the plates and transferred to a 2 mL reaction tube. 500 µL of chloroform (containing 2 µg/mL of heptadecanoic acid as an internal standard) were added, the tubes were shaken at 1200 rpm for 5 min, and centrifuged for 10 min at 20,000× *g* at 4 °C. A volume (200 µL) of the lower phase were evaporated in a vacuum concentrator, resuspended in 100 µL isopropanol/acetonitrile/water (2:1:1, *v*/*v*/*v*), and subjected to targeted LC/MS analysis. Lipids were normalized to an internal standard and to the sum of all lipids. Values were range-scaled and analyzed as well as visualized using MetaboAnalyst (version 6) (volcano plot analyses, *t*-test analysis, ANOVA, and heat map generation).

## Data Availability

The datasets generated and/or analyzed during the current study are available from the corresponding author on reasonable request.

## Supplementary Materials

The supporting information can be downloaded at: https://www.mdpi.com/article/10.3390/ijms26052272/s1.
