# Supplementary material for: Lectin-Based Substrate Detection in Fabry Disease Using the Gb3-Binding Lectins StxB and LecA
Source: Int J Mol Sci. 2025 Mar 4;26(5):2272. doi: 10.3390/ijms26052272 (PMC11900420; doi:10.3390/ijms26052272)
Supplement: Supplementary file 1 [file ijms-26-02272-s001.zip › ijms-3467450-supplementary.pdf]

# Lectin-based substrate detection in Fabry disease using the Gb3-binding lectins StxB and LecA

**Serap Elçin-Guinot[1, 2, 3], Simon Lagies[4, 5], Yoav Avi-Guy[1, 2, 3], Daniela Neugebauer[1, 2, 3], Tobias. B. Huber[6, 7], Christoph Schell[8, 9], Bernd Kammerer[2, 4, 5, 10] and Winfried Römer[1, 2, 3, 9, 10] ***

1 Faculty of Biology, University of Freiburg, Schänzlestraße 1, 79104 Freiburg, Germany

2 BIOSS, Centre for Biological Signaling Studies, University of Freiburg, Schänzlestraße 18, 79104 Freiburg, Germany

3 CIBSS, Centre for Integrative Biological Signaling Studies, University of Freiburg, Schänzlestraße 18, 79104 Freiburg, Germany

4 Core Competence Metabolomics, Hilde-Mangold-Haus, University of Freiburg, Habsburgerstraße 19, 79104 Freiburg, Germany

5 Institute of Organic Chemistry, University of Freiburg, Albertstraße 19, 79104 Freiburg, Germany

6 III. Department of Medicine and

7 Hamburg Center for Kidney Health, University Medical Center Hamburg-Eppendorf (UKE), Matinistraße 52, 20246 Hamburg, Germany

8 Faculty of Medicine, Institute for Surgical Pathology Medical Center, University of Freiburg, Breisacher Str. 115A, 70106 Freiburg, Germany

9 Freiburg Institute for Advanced Studies (FRIAS), University of Freiburg, Albertstraße 19, 79106 Freiburg, Germany

10 Spemann Graduate School of Biology and Medicine (SGBM), University of Freiburg, Albertstraße 19, 79104 Freiburg, Germany

***** Correspondence: Winfried Römer (winfried.roemer@bioss.uni-freiburg.de), Schänzlestraße 18, University of Freiburg, 79104 Freiburg, Germany

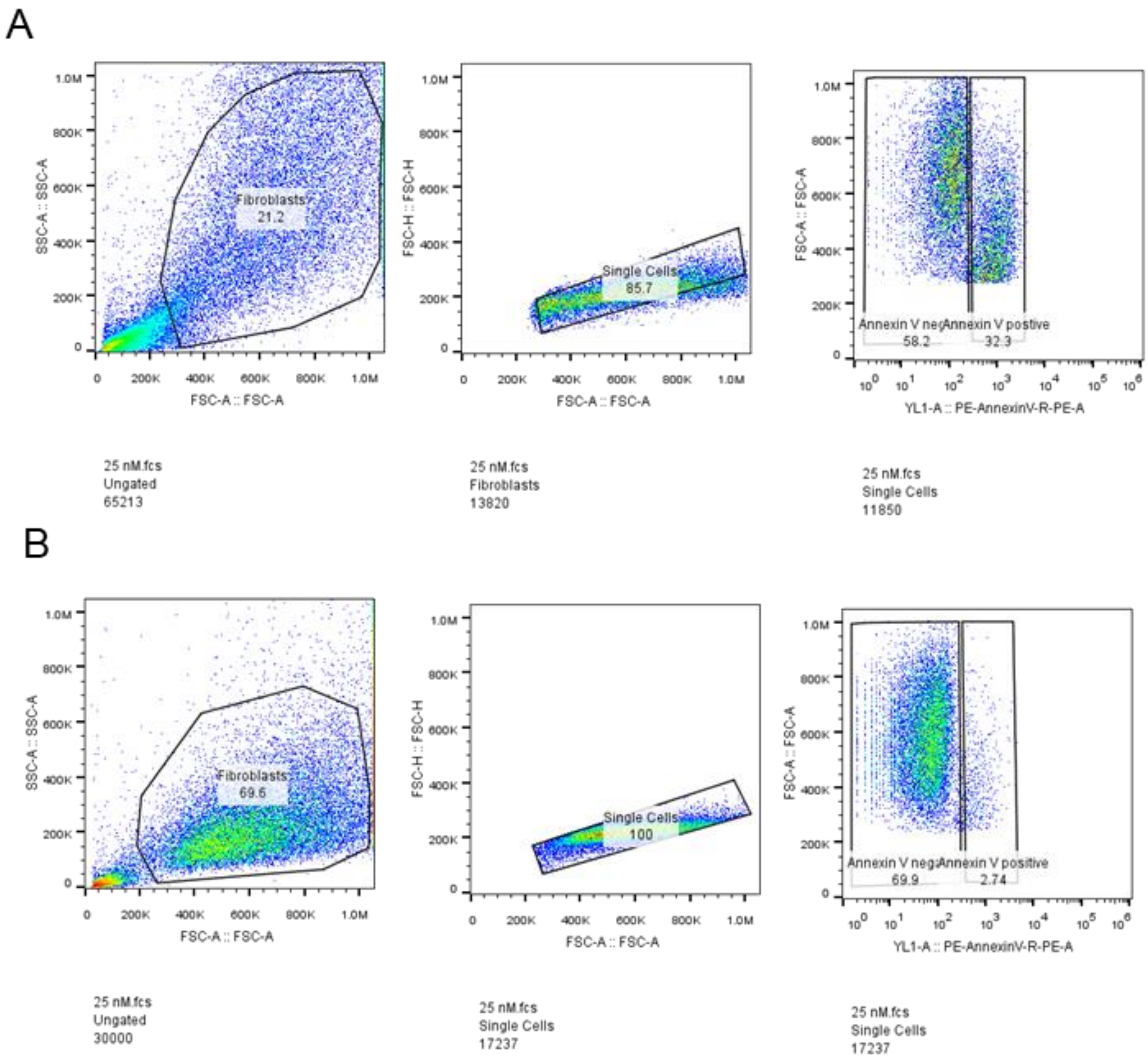


**Supplementary Figure S1: Gating strategy for Fibroblasts using FlowJo (A, left panel)** Healthy fibroblasts were scattered in the SSC-FSC graph and did not form a distinct population, unlike Fabry fibroblasts **(B, left panel)**. All fibroblasts were sub-gated to isolate single cells by excluding clusters, which were predominantly observed in healthy fibroblasts **(middle panels, A and B)**. Subsequently, all fibroblasts were incubated with Annexin V-PE to differentiate apoptotic cells from the non-apoptotic cell population **(right panels, A and B)**.

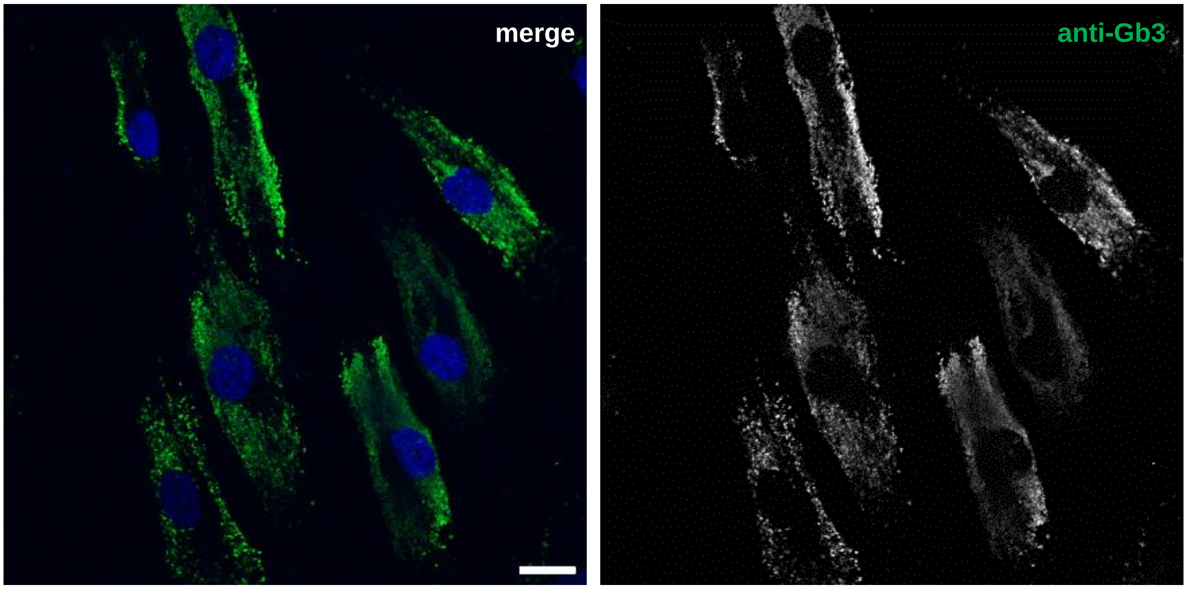


**Supplementary Figure S2: Anti-Gb3 Antibody Staining in Fabry Fibroblasts as a control experiment for the triple staining procedure.** The anti-Gb3 antibody was incubated individually as a single stain to validate the functionality of each Gb3-binding molecule. The anti-Gb3 antibody was indirectly labeled using a secondary anti-rat-AF 488 antibody. The left panel shows the anti-Gb3 staining in green, along with DAPI (blue) for nuclear staining. The right panel presents a grayscale representation of the anti-Gb3 signal. The scale bar represents 20 µm.

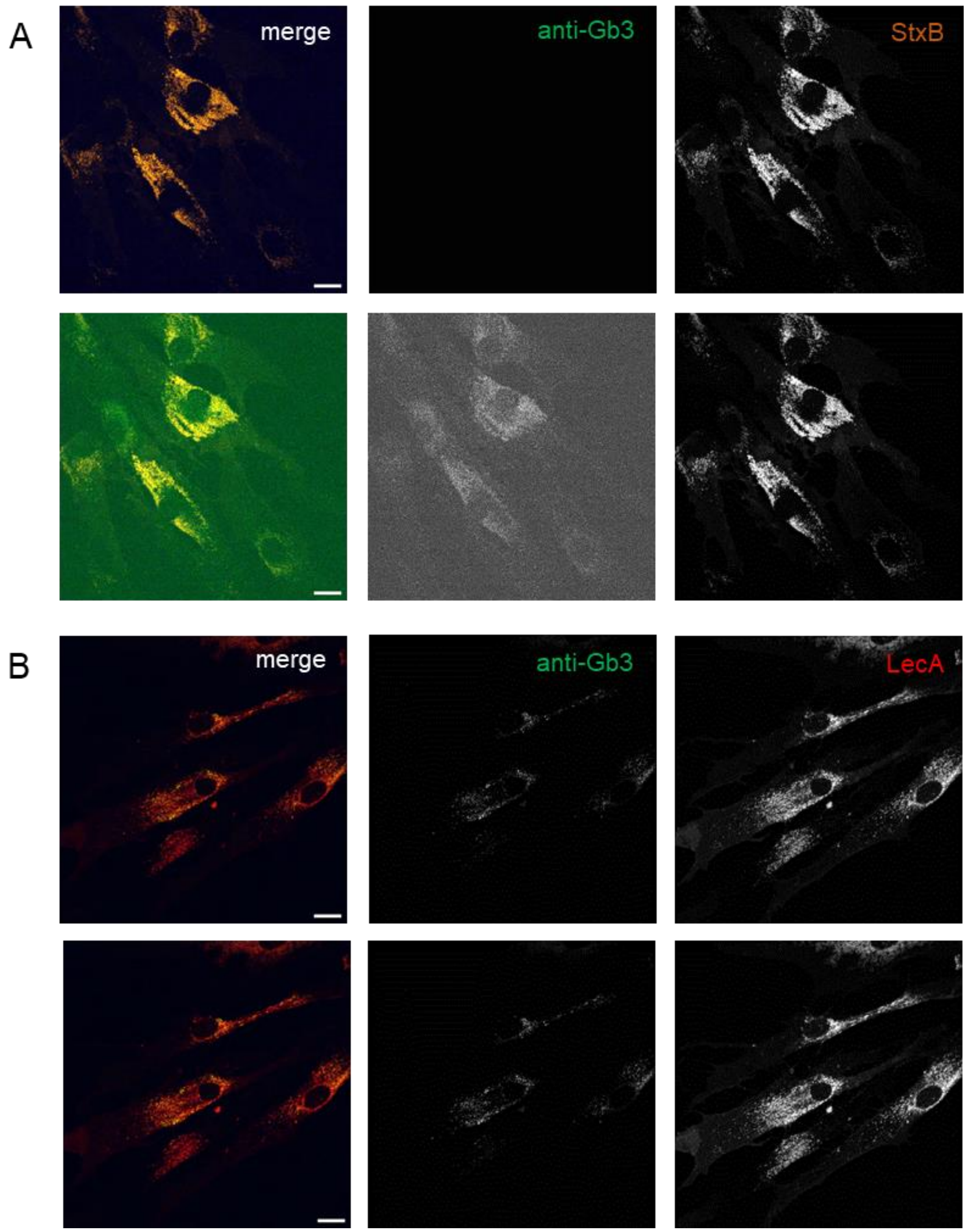
A
merge
anti-Gb3
StxB
B
merge
anti-Gb3
LecA

**Supplementary Figure S3: Double staining in Fabry fibroblasts without pre-incubation of anti-Gb3. (A)** Double-staining experiments were initially performed without pre-incubation of any individual Gb3-binding molecules to analyze their binding kinetics in double combinations. The anti-Gb3 antibody was indirectly labeled as previously. The upper panel shows that anti-Gb3 staining does not produce a detectable signal in the presence of StxB. **(B)** Pixel histograms in the lower panel were adjusted (stretched) to highlight the weak signal in the anti-Gb3 channel. The resulting image resembles the StxB signal, indicating bleed-through from the StxB channel. **(C)** Conversely, when LecA was used to stain cells along with the anti-Gb3 antibody, the anti-Gb3 staining produced a detectable signal with bright, concentrated vesicular structures (yellow arrowheads highlight these structures). This suggests that the anti-Gb3 antibody can bind to certain Gb3 molecules in Fabry fibroblasts in the presence of LecA. **(D)** The lower panel depicts histogram-stretched images, similar to those in panel B. Because of the existence of high levels of anti-Gb3 signals as such pointed by arrowheads, histogram-stretched image do not reflected the low signals as in panel B. All scale bars represent 20 µm.

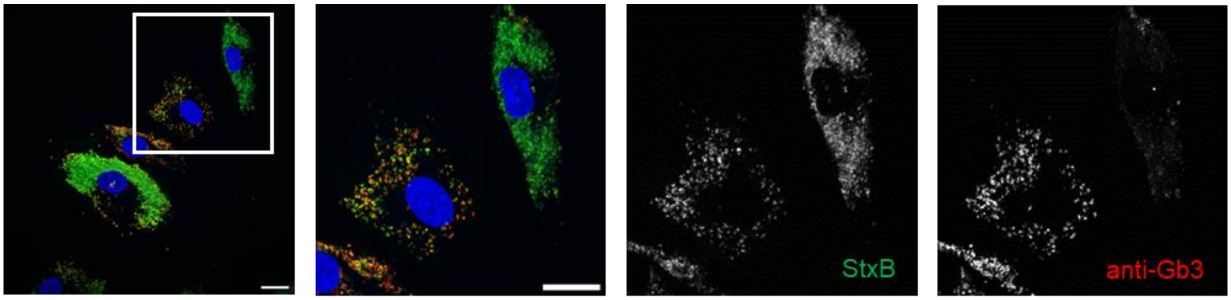


**Supplementary Figure S4: Heterogeneous staining of anti-Gb3 in Fabry fibroblasts.** The images show double-staining experiments performed with StxB and anti-Gb3 molecules. A combination of StxB (AF488) and anti-Gb3 (AF647, indirectly labeled) is depicted in colored and gray-scale images. In this experiment, the anti-Gb3 antibody was pre-incubated. Grayscale images clearly demonstrate that the anti-Gb3 antibody did not stain the rightmost cell. All scale bars represent 20 µm.

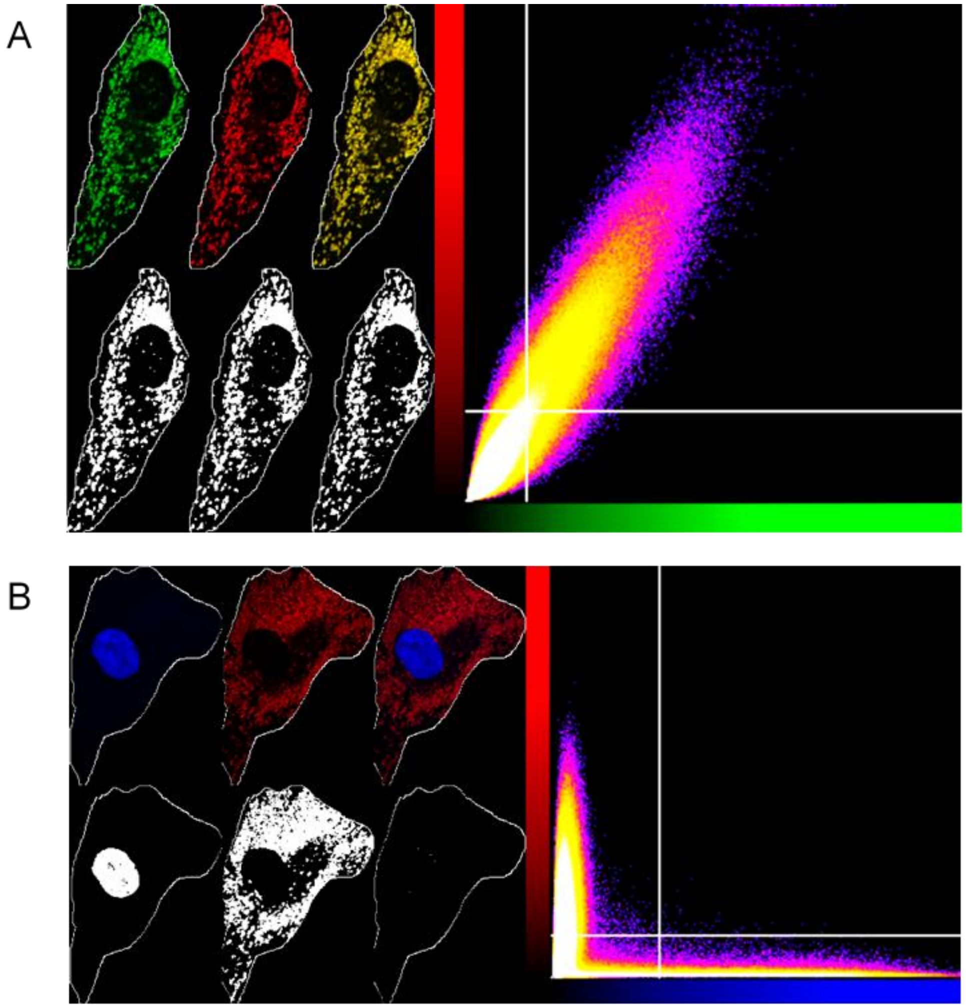


**Supplementary Figure S5: Experimental "Complete" and "No Overlap" cases calculated in Fabry fibroblasts using Manders Colocalization Coefficients (MCC, M1 & M2).** The primary Lamp1 antibody was indirectly labeled with anti-mouse AF 488 (green) and AF 647 (red) secondary antibodies. A complete overlap was simulated between the green and red channels (A), while a no-overlap scenario was simulated between the DAPI (blue) and red channel (B). The output images from the BIOP-JACoP plugin display binary thresholded images of the respective channels and scatter plots of pixel distributions for those channels is presented in A for complete case and in B for no overlap case. For the complete overlap case, M1 (the proportion of green pixels overlapping with red pixels) was 0.90, and M2 (the proportion of red pixels overlapping with green pixels) was 0.91. In contrast, for the no-overlap case, M1 (the proportion of blue pixels overlapping with red pixels) was 0.013, and M2 (the proportion of red pixels overlapping with blue pixels) was 0.2.

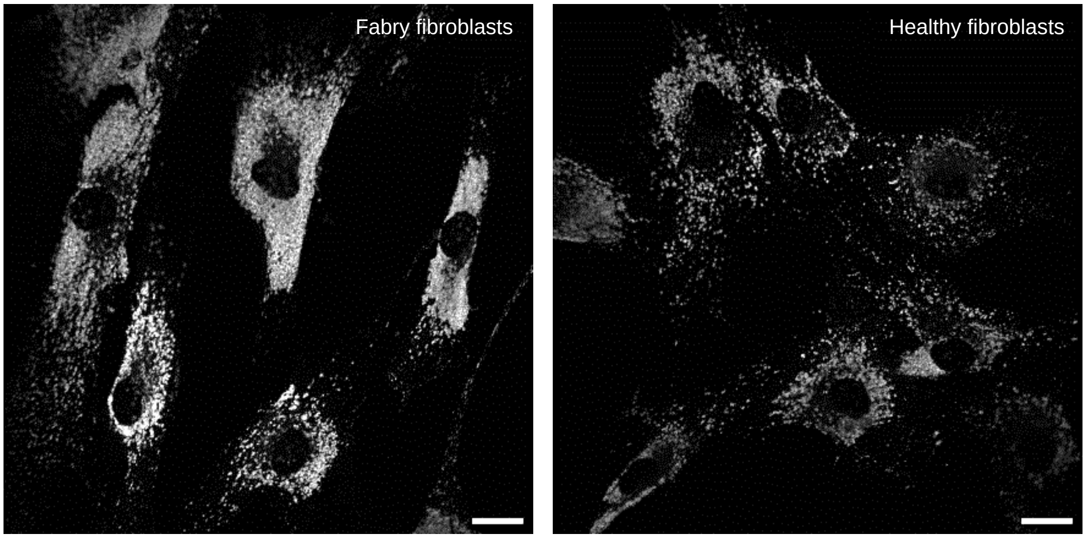


**Supplementary Figure S6: Lysosomal staining in Fabry and healthy fibroblasts.** Lysosomal structures in Fabry fibroblasts exhibit impaired volume and structural differences compared to their healthy counterparts. In healthy fibroblasts, Lamp1 antibody staining revealed vesicular structures. In contrast, lysosomal structures in Fabry fibroblasts appear heavily concentrated, making individual vesicular structures less distinguishable. Additionally, the overall signal intensity was markedly increased in Fabry fibroblasts compared to healthy fibroblasts. All scale bars represent 20 µm.

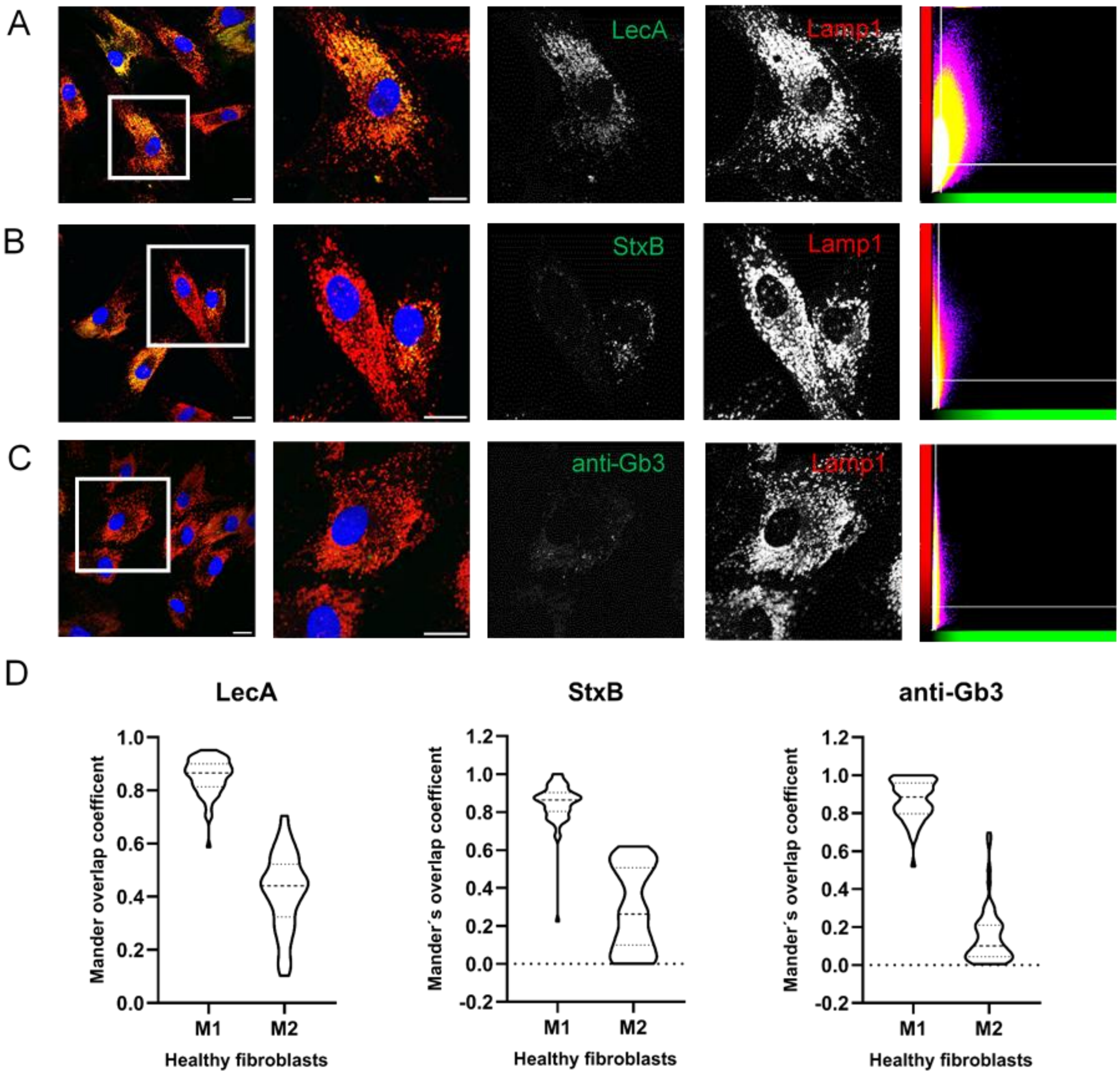
A
LecA
Lamp1
B
StxB
Lamp1
C
anti-Gb3
Lamp1
D
LecA
Mander overlap coefficent
1.0
0.8
0.6
0.4
0.2
0.0
M1
M2
Healthy fibroblasts
StxB
Mander´s overlap coefficent
1.2
1.0
0.8
0.6
0.4
0.2
0.0
-0.2
M1
M2
Healthy fibroblasts
anti-Gb3
Mander´s overlap coefficent
1.2
1.0
0.8
0.6
0.4
0.2
0.0
-0.2
M1
M2
Healthy fibroblasts

**Supplementary Figure S7: Lysosomal staining in combination with LecA-AF488, StxB-AF488 and antiGb3 in healthy fibroblasts.** Confocal images showing healthy fibroblasts (all panels), stained with anti-Lamp1 (indirectly labeled with anti-mouse AF647 antibody). Cells are counter stained with LecA-AF488 **(A)**, StxB-AF488 **(B)**, and with anti-Gb3 (indirectly labeled with anti-rat AF488 antibody). **(C)** All panels are also represented as zoomed area of full view (2048 x 2048 pixels) and as gray scale images from each channel from left to right. Scatter plots of pixels from individual channels are demonstrated from the zoomed area (most right). **(D)** Signals arising from indicated labels were recorded from each cell as z-stacks for a 3-dimentional colocalization analysis using BIOP-JACOP plugin in ImageJ (n ≥ 60 cells). MCC was used to evaluate the overlapping signals from individual channels to calculate colocalization using a pixel-based colocalization method. Cells were manually thresholded to remove background signals. M1 indicates the fraction of overlapping pixels from green channel in red channel, and M2 is vice versa. MCC values range from 0 to 1.0, whereby 1.0 indicates perfect colocalization. MCC values are represented using a violin plot, which illustrates the median, interquartile range, outliers, and the probability density of the data. Wider sections of the plot indicate areas with higher data density, while narrower sections represent lower density regions. All scale bars are 20 µm.

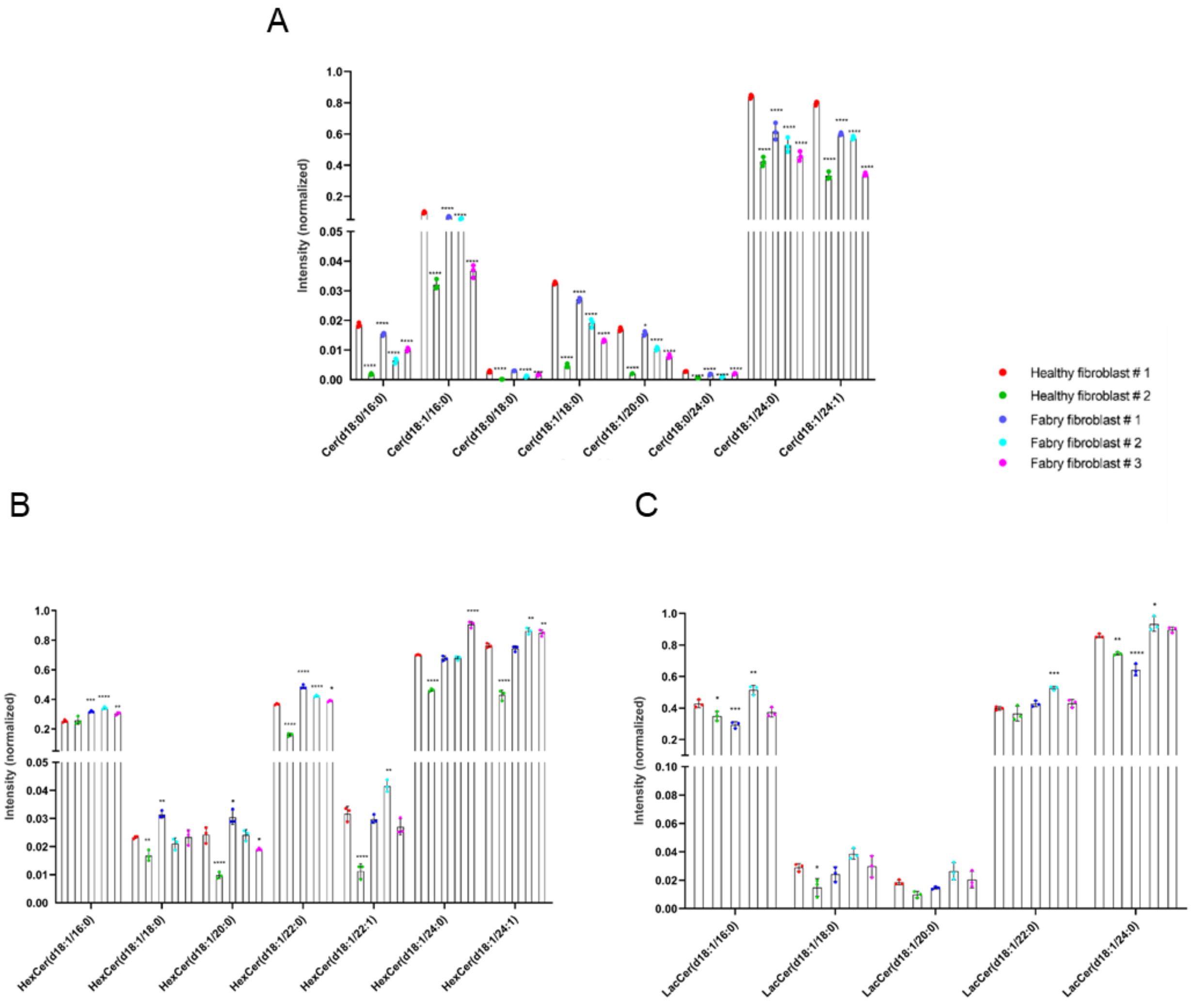


**Supplementary Figure S8: MS Analysis of fibroblasts assessing precursors of Gb3.** Intensity peaks representing the amounts of Cer (A), HexCer (B), and LacCer (C) isoforms in Fabry and healthy fibroblasts were measured using targeted LC/MS analysis. Data are presented as means, with error bars indicating SD. Statistical significance of the differences was tested using one-way ANOVA followed by Dunnett's multiple comparisons test. Only significant values are shown. $^*p < 0.05$, $^{**}p < 0.01$, $^{***}p < 0.001$, $^{****}p < 0.0001$.

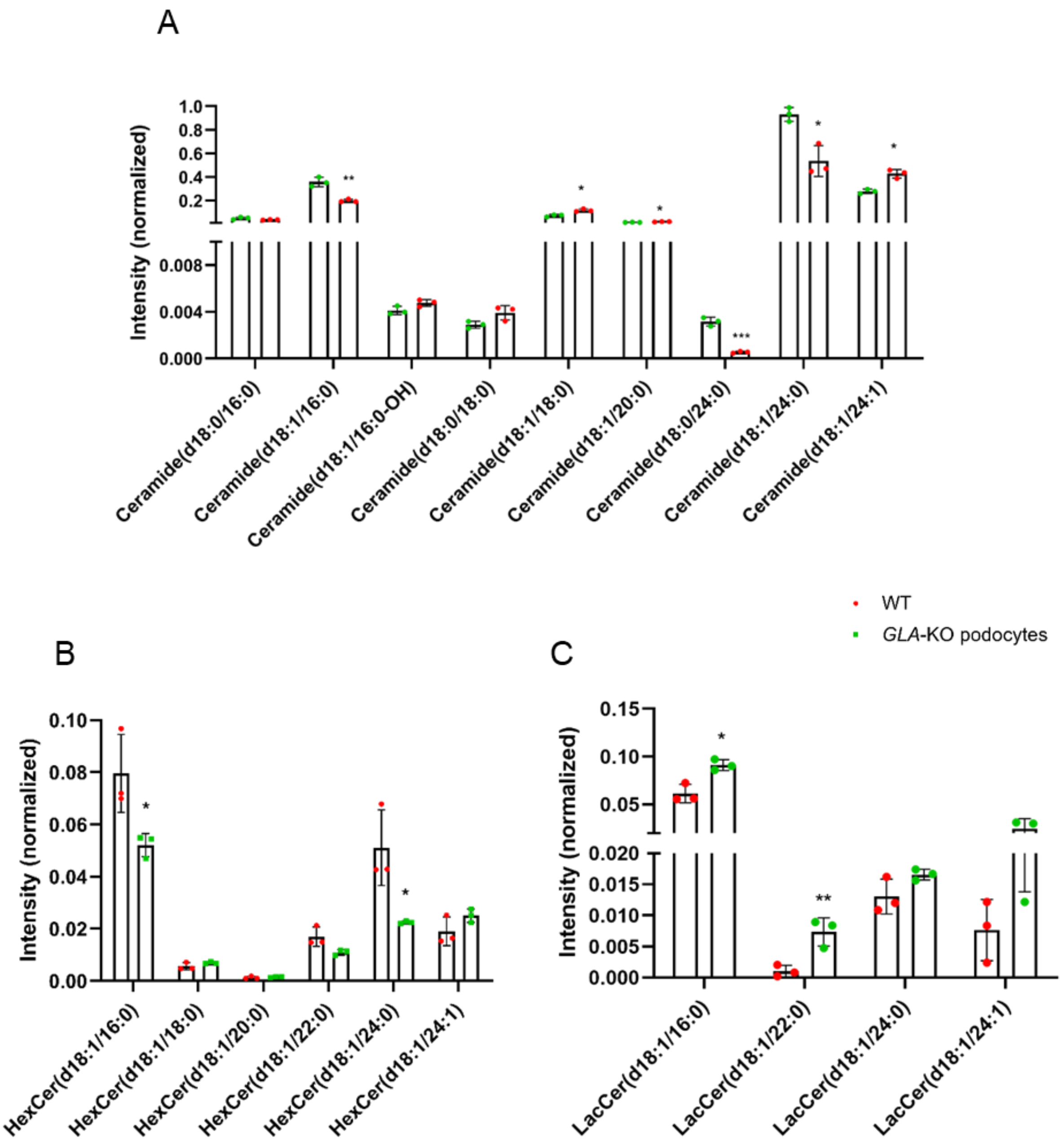


**Supplementary Figure S9: MS Analysis of podocytes assessing precursors of Gb3.** Intensity peaks representing the amounts of Cer (A), HexCer (B), and LacCer (C) isoforms in *GLA*-KO podocytes and wild-type podocytes were measured using targeted LC/MS analysis. Data are presented as means, with error bars indicating SD. Statistical significance of the differences was tested using an unpaired two-tailed t-test. Only significant values are shown. *p < 0.05, **p < 0.01, ***p < 0.001, ****p < 0.0001.
